# Unveiling the Functions of Two RpoNs in *Bradyrhizobium* sp. DOA9 During Free-Living Conditions: A Comprehensive and Comparative Analysis

**DOI:** 10.3390/ijms27104304

**Published:** 2026-05-12

**Authors:** Jenjira Wongdee, Teerana Greetatorn, Pongdet Piromyou, Pongpan Songwattana, Natcha Pruksametanan, Neung Teaumroong, Nantakorn Boonkerd, Pakpoom Boonchuen, Eric Giraud, Panlada Tittabutr

**Affiliations:** 1 Institute of Research and Development, Suranaree University of Technology, Nakhon Ratchasima 30000, Thailand; jenjira.w@sut.ac.th (J.W.); teerana@sut.ac.th (T.G.); pongdet.p@sut.ac.th (P.P.); pongpan.s@sut.ac.th (P.S.); 2 School of Biotechnology, Institute of Agricultural Technology, Suranaree University of Technology, Nakhon Ratchasima 30000, Thailand; natcha.pruksametanan@gmail.com (N.P.); neung@sut.ac.th (N.T.); nantakon@sut.ac.th (N.B.); pakpoom.b@sut.ac.th (P.B.); 3 IRD, Plant Health Institute of Montpellier, UMR-PHIM, 113, IRD/CIRAD/INRAE/Université de Montpellier/SupAgro, Campus de Baillarguet, TA-A82/J, 34398 Montpellier Cedex 5, France; eric.giraud@ird.fr; 4 Graduate School of Life Science, Tohoku University, Sendai 980-8577, Japan

**Keywords:** *rpoN*, *Bradyrhizobium*, free-living conditions, transcriptome, nitrogen fixation, regulation

## Abstract

In this study, we investigate two RpoN homologs in *Bradyrhizobium* sp. DOA9—chromosomal RpoNc and megaplasmid-borne RpoNp—and their roles in free-living conditions and nitrogen fixation. Phylogenetic analysis showed that RpoNc clusters with RpoN proteins from symbiotic nitrogen-fixing strains, whereas RpoNp forms a distinct clade, consistent with a function in stress responses. RpoNc proved essential for free-living conditions: Δ*rpoNc* mutants displayed severe growth defects that RpoNp could not compensate for. Transcriptomic comparisons between wild type and mutant RpoN identified 541 differentially expressed genes (DEGs) grouped into three clusters: 100 downregulated, 175 upregulated, and 254 moderately downregulated (with a fold change > 2, and a q-value (FDR, padj) < 0.05). Affected pathways involved nitrogen metabolism, motility, and environmental adaptation. RpoNc controlled major nitrogen fixation genes (*nif and fix*) along with core growth and stress response functions, while RpoNp mainly influenced stress-adaptation pathways. Genome-wide promoter motif analysis predicted 68 putative RpoNc targets, mainly associated with nitrogen fixation and metabolism, compared with only 22 predicted RpoNp targets, indicating a more restricted regulon. Electrophoretic mobility shift assays (EMSAs) further confirmed that both RpoN proteins directly bind σ^54^-dependent promoters identified from transcriptomic data, supporting their regulatory roles under free-living conditions. Two mutants (Δ*rpoNc* and Δ*rpoNp*::Ω*rpoNc*) showed broad transcriptional disruption across nitrogen fixation, metabolism, and stress responses, underscoring complementary regulation. Overall, RpoNc is the dominant regulator of nitrogen fixation and core metabolism during free-living conditions, whereas RpoNp fine-tunes stress responses, revealing new regulatory insights for DOA9 adaptation. These results clarify how RpoN systems optimize survival across fluctuating conditions.

## 1. Introduction

Nitrogen-fixing bacteria, such as *Bradyrhizobium*, play a crucial role in supporting plant growth by converting atmospheric nitrogen (N_2_) into bioavailable ammonium through the action of the nitrogenase enzyme complex [1]. This process is tightly regulated by various genetic and environmental factors to ensure its efficiency and adaptability. Among the key regulators, the alternative sigma factor RpoN (σ^54^) is essential for the transcription of nitrogen fixation (*nif*) genes and other metabolic pathways crucial for bacterial survival and symbiotic efficiency [2]. In many nitrogen-fixing bacteria, including *Bradyrhizobium*, the interplay between RpoN and the transcriptional activator NifA governs the expression of *nif* genes, ensuring optimal nitrogen fixation in response to environmental cues. Additionally, RpoN is involved in a wide array of cellular processes, including motility, carbon metabolism, biofilm formation, stress responses, and virulence, which are critical for bacterial adaptation in diverse environments [3].

The RpoN regulatory system is essential for controlling the transcription of numerous bacterial genes, enabling bacteria to adapt to varying environmental conditions and host interactions. RpoN directs RNA polymerase (RNAP) to the conserved -12 (TGC) and -24 (GG) promoter elements, which are part of the broader consensus sequence YTGGCACGrNNNTTGCW [4,5]. This interaction results in the formation of a closed complex, which is energetically stable and infrequently transitions into the open complex. RpoN-dependent transcription is a requirement for bacterial enhancer-binding proteins (bEBPs), which bind to upstream activator sequences (UASs) located approximately 80 to 150 bp upstream of the core promoter [6,7]. These bEBPs assist in forming the open complex by interacting with the σ^54^-RNA polymerase holoenzyme, often through DNA looping, which brings the bEBP in proximity to the promoter-bound holoenzyme [8,9]. For example, in the regulation of natural product gene clusters in *Myxococcus xanthus*, mutations in the UASs of the enhancer-binding protein Nla28 can significantly decrease promoter activity, underscoring the vital role of precise bEBP–DNA interactions in σ^54^-dependent transcriptional regulation [10].

The role of RpoN extends beyond nitrogen fixation and varies across different bacterial species. For example, in *Escherichia coli*, RpoN regulates nitrogen assimilation, flagellar biosynthesis, and stress responses [1], whereas in *Pseudomonas aeruginosa*, it controls virulence, quorum sensing, and biofilm formation, and also contributes to antibiotic stress survival [11]. Also, in *P. fluorescens*, *rpoN* deletion leads to reduced motility and biofilm formation, underscoring its role in bacterial adaptability [12]. In *Rhizobium etli*, RpoN plays a role in nitrogen metabolism and motility but is not essential for symbiotic nitrogen fixation. In contrast, *B. diazoefficiens* USDA110, which contains two copies of *rpoN*, requires both for full nitrogen-fixing capacity in symbiosis with soybean [13]. 

*Bradyrhizobium* sp. strain DOA9 is a unique diazotrophic bacterium capable of fixing nitrogen under both free-living and symbiotic conditions with *Aeschynomene americana*. This strain carries two copies of *rpoN* located on the chromosome (*rpoNc*) and symbiotic plasmid (*rpoNp*), together with two *nifA* genes, indicating a complex regulatory network controlling nitrogen fixation and cellular metabolism [14,15]. Previous studies showed that both chromosomal and plasmid-borne *nif* genes contribute to nitrogenase activity during symbiosis, whereas only chromosomal *nif* genes are required under free-living conditions [14], leaving the roles of the two RpoN proteins in free-living regulation unclear. Genomic analysis further revealed that *rpoNc* is positioned between *lptCA* and *hpf–ptsN*, linking it to lipopolysaccharide transport and carbon–nitrogen metabolism. Although RpoNc (541 aa) and RpoNp (553 aa) share 55.82% identity and conserve the σ^54^ domains, RpoNp exhibits large deletions between an N-terminal activator interacting domain and core enzyme binding domain and a C-terminal extension, suggesting functional divergence between the two regulators [15].

Given the multifaceted role of RpoN in bacterial physiology, this study aims to comprehensively analyze the transcriptomic profiles of genes in response to RpoNc and RpoNp in *Bradyrhizobium* sp. DOA9 under free-living conditions. Additionally, we examined the impact of single and double *rpoN* mutations on the expression of *nif* and other metabolic genes to determine their distinct or overlapping regulatory roles. To validate the RpoN-regulated genes identified from the transcriptomic data, we further investigated the DNA-binding interactions of RpoNc and RpoNp with upstream regions of the target genes using an Electrophoretic Mobility Shift Assay (EMSA). The influences of two RpoNs on carbon and nitrogen assimilation, extracellular polysaccharide (EPS) production, and biofilm formation—which are essential for bacterial adaptation and survival—were also investigated. Our findings provide critical insights into the functional divergence and regulatory crosstalk between the two RpoN homologs of strain DOA9, deepening our understanding of their contributions to bradyrhizobial physiology and free-living capacity in an environment lacking legume partners for symbiosis. 

Together, these findings position DOA9 as a unique model for understanding sigma factor diversification in *Bradyrhizobium* and reveal previous unrecognized layers of σ^54^-dependent control in this strain that coordinate survival, metabolic flexibility, and nitrogen-fixing efficiency under microaerobic conditions. This expanded regulatory landscape substantially advances our understanding of RpoN biology and offers new insight into how free-living nitrogen-fixing bacteria integrate environmental sensing with core metabolic programs.

## 2. Results

### 2.1. Phylogenetic Analysis of RpoN in Bradyrhizobium Strains

**Figure 1:**
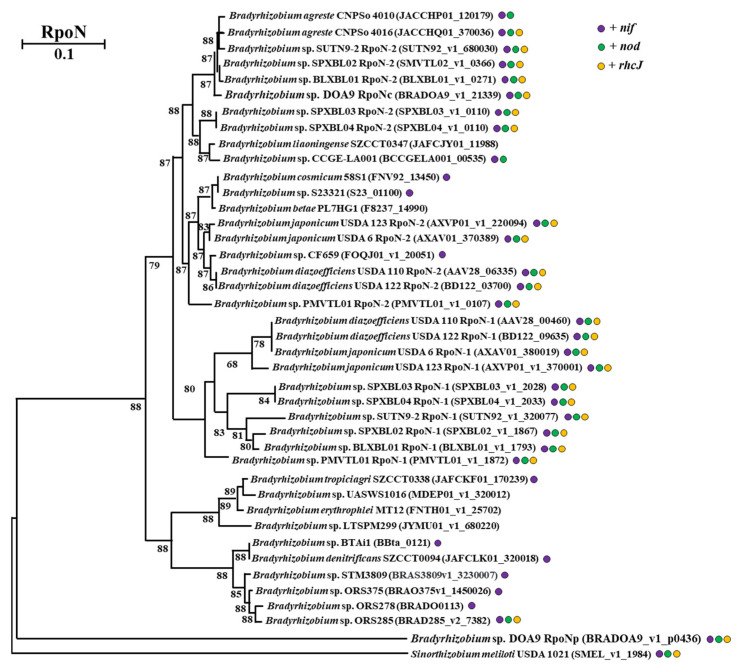
Phylogenetic tree of RpoN proteins from various *Bradyrhizobium* strains constructed using the maximum-likelihood method with 1000 bootstrap replicates. Colored dots placed after each strain name indicate the presence of symbiosis-related genes. Strains marked with three colored dots possess *nif* (purple dot) and *nod* (green dot) genes and also contain the Type III secretion system gene *rhcJ* (yellow dot). Strains without any dot lack *nif*, *nod* and *rhcJ* genes. The RpoN protein from *Sinorhizobium meliloti* USDA 1021 was used as the outgroup.

The phylogenetic analysis of RpoN in *Bradyrhizobium* strains revealed that *Bradyrhizobium* sp. DOA9 possesses two distinct RpoN proteins. The phylogenetic tree in Figure 1 illustrates the evolutionary relationships among the *Bradyrhizobium* strains, based on the presence of *nif* and *nod* genes, as well as a Type III Secretion System gene (*rhcJ*), in their genomes. Notably, strains harboring these genetic elements (indicated as strains with the three colored dots in the tree) exhibited distinct clustering patterns. The chromosomal RpoN protein of DOA9 (RpoNc) clusters with RpoN proteins from other strains known to regulate symbiotic nitrogen fixation and nodulation, including SUTN9-2, SPXBL-02, BLXBL-01, SPXBL-03, and SPXBL-04. There are also *Bradyrhizobium* sp. strains possessing two copies of RpoN, similar to DOA9. RpoNc was grouped in RpoN copy2 of this clade, whereas RpoN copy1 was separated and distributed in other positions. This clustering pattern is consistent with the theory that the chromosomally encoded RpoN (RpoNc) is associated with regulatory functions in nitrogen metabolism and symbiosis-associated processes during host–plant interactions. In contrast, the plasmid-encoded RpoN (RpoNp) of DOA9 forms a distinct outgroup, which may reflect functional divergence and is potentially linked to the regulation of other metabolic pathways.

Furthermore, a correlation was observed between specific nodulation phenotypes and the presence of RpoN copy 1, particularly in strains capable of nodulation with soybean, USDA strains, and the broad-host-range strain SUTN9-2, along with members of the predicted soybean-nodulating group from Laos, LDR [16]. Photosynthetic *Bradyrhizobium* strains closely related to *B. denitrificans* SZCCT0094 were suggested to cluster with strains exhibiting efficient nitrogen fixation under free-living conditions. Notably, the RpoN proteins of the free-living strains 58S1, S23321, and CF659, which carry only the *nif* gene (represented by a purple dot in the tree), clustered with RpoN copy2 of the USDA strains and with the free-living strain PL7HG1, which lacks the *nif*, *nod*, and *rchJ* genes (without dot in the tree).

This phylogenetic separation of RpoN proteins into distinct clusters indicates functional diversification, with one RpoN variant likely serving as a generalist sigma factor regulating a broad range of genes, and the other functioning as a specialist that activates specific genes under certain conditions. Additional transcriptomic analysis under free-living conditions may further clarify the regulatory roles of these two RpoN proteins in DOA9 and provide deeper insights into their functional specialization.

### 2.2. Chromosomal RpoN Is Essential for Free-Living Conditions of Bradyrhizobium sp. DOA9

**Figure 2:**
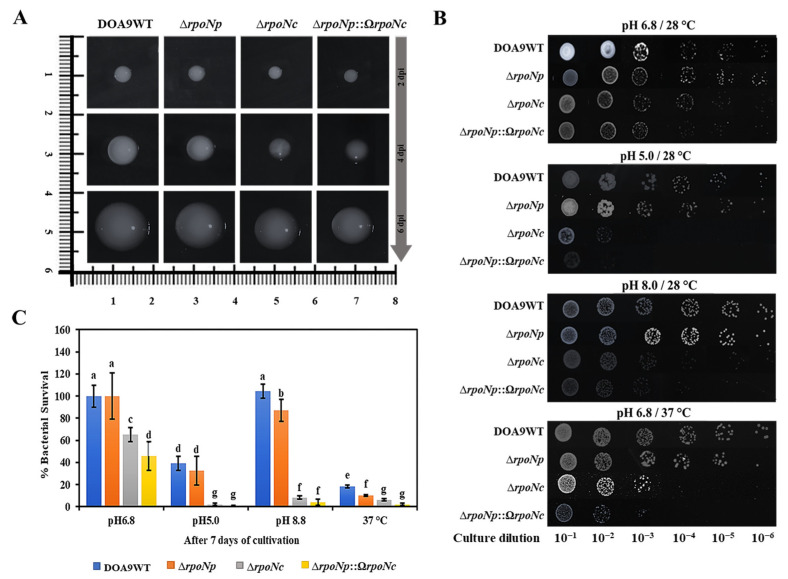
(**A**) Colony morphology comparison of DOA9WT and its derivatives observed at 2, 4, and 6 days on YEM plates under a microscope (Axes are in centimeters (cm)). (**B**) Bacterial growth was measured in colony forming units (CFU) over a 7-day period and (**C**) bacterial survival (%) was determined using the drop-plate technique under both normal growth conditions (pH 6.8 at 28 °C) and stress conditions (acidic pH 5, alkaline pH 8 at 28 °C, and pH 6.8 at 37 °C). Different letters indicate statistically significant differences (*p* < 0.05).

The chromosome-encoded RpoNc plays a critical and nonredundant role in supporting the free-living conditions of *Bradyrhizobium* sp. DOA9. Morphological and biochemical analyses have consistently demonstrated that RpoNc functions as the principal sigma factor regulating growth-related processes in this bacterium [15]. A comparative colony morphology analysis further confirmed its role: the ∆*rpoNc* and ∆*rpoNp*::Ω*rpoNc* mutants displayed distinct aberrant phenotypes. When colony morphology was examined on YM agar, the strain lacking RpoNc exhibited a markedly reduced colony size compared with the wild type (WT) and ∆*rpoNp* strains from day 2 to day 6 of incubation. Notably, its function could not be compensated for by the plasmid-encoded RpoNp in the ∆*rpoNp*::Ω*rpoNc* mutant, as was evident from their distinct colony phenotypes (Figure 2A). Bacterial viability assays, including serial dilution using the drop plate method and survival percentage tests, showed a severe loss of survival capacity for both the ∆*rpoNc* and ∆*rpoNp*::Ω*rpoNc* mutants, affected by different growing conditions at normal, acidic, and alkaline pH and 37 °C. In contrast, the WT and ∆*rpoNp* strains exhibited only minor differences in growth and viability (Figure 2B,C). These findings underscore the essential role of RpoNc in maintaining normal physiological functions and cellular viability under free-living conditions. Although RpoNc is clearly indispensable, the data also suggest that RpoNp contributes modestly to free-living conditions. This is reflected in the phenotypic outcomes of the double *rpoN* mutant, where the additional deletion of *rpoNp* caused only limited changes across the results from multiple assays [15].

### 2.3. Transcriptomic Profiling of RpoN-Regulated Genes in Bradyrhizobium sp. DOA9

To investigate the potential regulation of gene expression by the two RpoN proteins in *Bradyrhizobium* sp. DOA9, the transcriptomic data from wild type bacteria compared to that of ∆*rpoNc* and ∆*rpoNp*::Ω*rpoNc* mutant strains growing under microaerobic and nitrogen free conditions were analyzed. The principal component analysis (PCA) showed clear separation of wild type DOA9 from both mutants in PC2, which accounted for 15.7% of the variance, indicating that major transcriptional differences were represented on this axis (Figure S1). In contrast, PC1 explained the largest portion of variance (43.2%) but did not correspond to strain-specific clustering.

**Figure 3:**
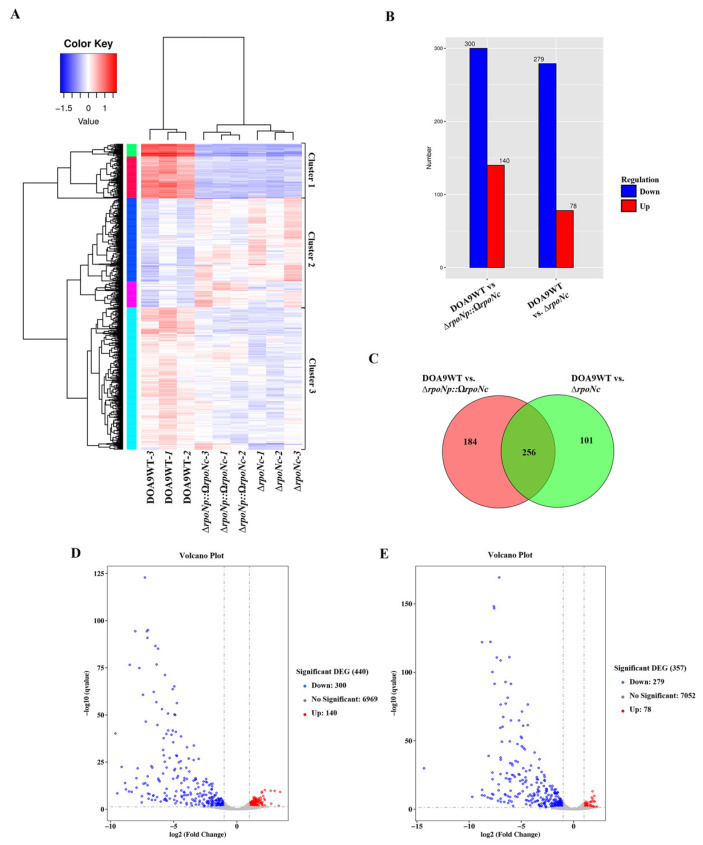
(**A**) Cluster analysis of differentially expressed genes based on Log10(FPKM + 1) values. Hierarchical clustering, using FPKM values under varying experimental conditions, generates a dendrogram that highlights clusters of genes with shared functional roles and biological processes. Numbers appended to the sample IDs indicate replicate numbers. Colors indicate functional categories: green, cellular metabolism and protein homeostasis; red, general cellular metabolism and transport; dark blue, central cellular processes including metabolism, regulation, and genetic information processing; pink, general cellular regulation, and metabolism-related functions; blue, gene expression, and cellular processes. (**B**) Bar graph displaying significantly up- and downregulated genes between groups. Differential expression analysis was conducted using DESeq2, identifying genes with a fold change > 2 and a *q*-value (FDR, padj) < 0.05. Upregulated genes are represented in red, while downregulated genes are shown in blue. (**C**) Venn diagram illustrating differentially expressed genes, showing unique and overlapping genes across different groups. Volcano plots of differential gene expression comparisons: (**D**) WT vs. ∆*rpoNc* and (**E**) WT vs. ∆*rpoNp::ΩrpoNc*. Red dots indicate significantly upregulated genes, while blue dots denote significantly downregulated genes. The *x*-axis represents the log2 fold change in gene expression, and the *y*-axis displays the statistical significance as log10(*q*-value, FDR, padj).

The total of 541 differentially expressed genes (DEGs) were identified using a threshold of fold change > 2 and *q*-value (FDR, padj) < 0.05, and were grouped into three clusters by hierarchical clustering (Figure 3A; Supporting Information Table S1). Cluster 1 comprised 100 strongly downregulated genes (green and red), Cluster 2 contained 175 upregulated genes (dark blue and pink), and Cluster 3 included 254 moderately downregulated genes (light blue) relative to Cluster 1. DESeq2 analysis further identified statistically significant DEGs, revealing 78 up- and 279 downregulated genes in DOA9WT vs. ∆*rpoNc*, and 140 up- and 300 downregulated genes in DOA9WT vs. ∆*rpoNp*::Ω*rpoNc* (Figure 3B). These results are visualized in volcano plots, where red and blue dots indicate significantly up- and downregulated genes, respectively (Figure 3D,E). A Venn diagram was used to visualize shared and unique DEGs between the two comparisons (Figure 3C). Of the 541 DEGs, 256 genes were common to both comparisons, whereas 101 genes were unique to DOA9WT vs. ∆*rpoNc*. In contrast, 184 DEGs were specifically identified in DOA9WT vs. ∆*rpoNp*::Ω*rpoNc* (WT vs. DB), reflecting the combined deletion of *rpoNc* and disruption of *rpoNp* in the DOA9 background. Several uniquely up- and downregulated genes in this set are therefore presumed to be co-regulated by RpoNp under free-living conditions through both positive and negative regulatory mechanisms, which is supported by our RT-qPCR validation.

**Figure 4:**
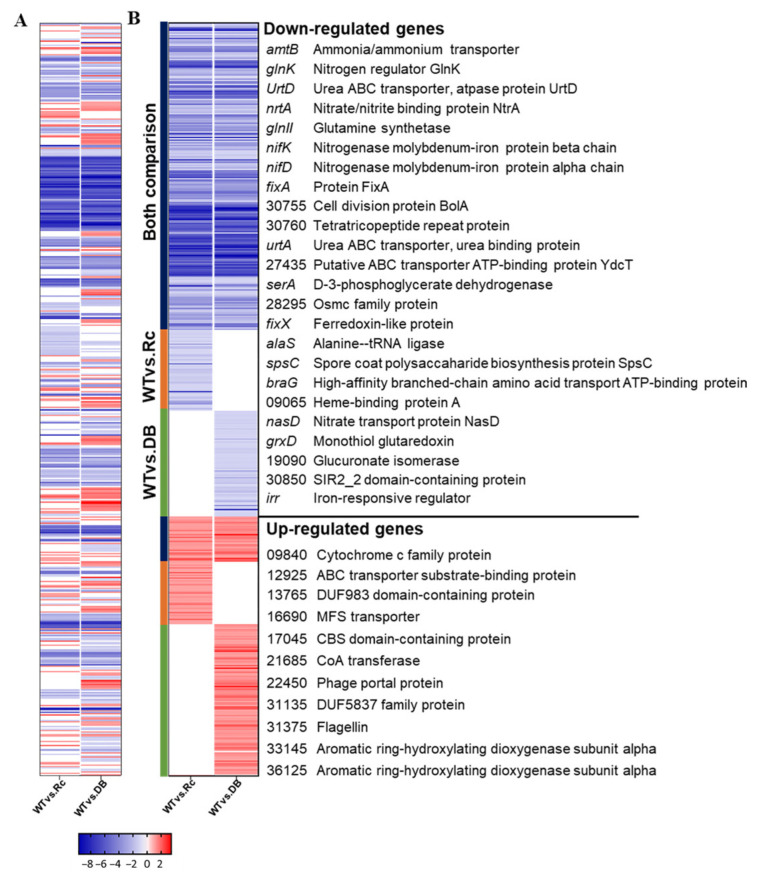
(**A**) Overview of 541 differentially expressed genes from the Venn diagram, illustrating the overlap of genes with significant differential expression (FDR < 0.05 and LFC > 2) across two comparisons: WT vs. ∆*rpoNc* and WT vs. ∆*rpoNp::ΩrpoNc*. (**B**) Heatmap displaying the expression patterns of 541 differentially expressed genes in both ∆*rpoNc* and ∆*rpoNp*::Ω*rpoNc* mutants compared to DOA9WT. The heatmap categorizes differentially expressed genes based on their presence in specific comparisons: genes found in both WT vs. ∆*rpoNc* and WT vs. ∆*rpoNp::ΩrpoNc* (both comparisons shown as blue line), genes unique to WT vs. ∆*rpoNc* (WT vs. Rc shown as orange line), and genes unique to WT vs. ∆*rpoNp::ΩrpoNc* (WT vs. DB shown as green line). The color-coded scale bars below the heatmap indicate normalized expression levels and log fold change (LFC). The right panel highlights a selection of key differentially expressed genes in the comparison of WT vs. ∆*rpoNc* and WT vs. ∆*rpoNp::ΩrpoNc*, with gene accessions represented in the BDOA9_RSxxxxx format.

**Figure 5:**
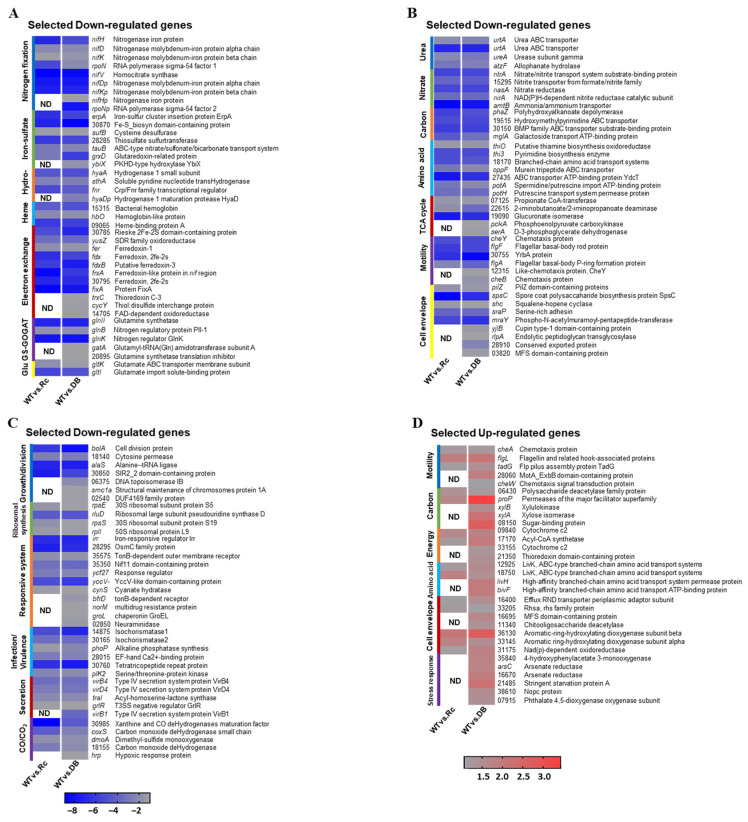
Lists of differentially transcribed genes categorized based on predicted function, with fold change represented by colored boxes corresponding to the scale in the heatmap. (**A**–**C**) display representative downregulated genes, while (**D**) shows representative upregulated genes. These genes were identified from differential expression analysis in the pairwise comparisons of WT vs. ∆*rpoNc* (WT vs. Rc) and WT vs. ∆*rpoNp*::Ω*rpoNc* (WT vs. DB).

To further examine RpoN-dependent regulation, DEGs were classified by comparison group using a heatmap (Figure 4): genes shared between both comparisons (blue), genes unique to DOA9WT vs. ∆*rpoNc* (orange), and genes unique to DOA9WT vs. ∆*rpoNp*::Ω*rpoNc* (green). Among the 256 shared genes, 225 were downregulated and 31 were upregulated, indicating that chromosomal RpoNc functions as both an activator and repressor under free-living conditions. Gene enrichment analysis revealed that downregulated genes were significantly enriched in nitrogen metabolism, cell motility, growth, two-component regulatory systems, and symbiotic infection, whereas upregulated genes were associated with carbohydrate metabolism, secondary metabolite biosynthesis, energy production, and amino-acid transport (Figure 4; Table S1). KEGG enrichment of the most strongly repressed genes (log2FC −12 to −9) highlighted pathways essential for nitrogen fixation, including urea utilization, nitrate assimilation, the GS–GOGAT cycle, electron transfer, ABC transport, and surface polysaccharide biosynthesis. In contrast, fewer upregulated genes showed modest fold changes and were mainly related to motility, carbon and energy metabolism, amino-acid metabolism, cell-envelope biosynthesis, and stress responses (Figure S2 and Figure 5D).

Across the transcriptomes, only 19 downregulated and 13 upregulated DEGs were located on the DOA9 plasmid, whereas the majority (503 DEGs) were chromosomally encoded, indicating that chromosomal RpoNc acts as the primary regulator of transcriptional responses under free-living conditions. Heatmap profiles supported this trend, revealing widespread downregulation in both comparisons and a subset of DEGs unique to the WT vs. Δ*rpoNp*::Ω*rpoNc* dataset. This pattern suggests that plasmid-encoded RpoNp contributes to transcriptional regulation, but to a lesser extent than RpoNc (Table S1; Figure 5).

To assess the functional significance of the DEGs, Gene Ontology (GO) enrichment analysis classified annotated genes into biological process, cellular component, and molecular function categories (Figure 5). Representative genes were selected for the heatmap based on key pathway roles and differential expression across transcriptomic comparisons. Expression patterns differed between WT vs. Δ*rpoNc* and WT vs. Δ*rpoNp*::Ω*rpoNc*; most genes showed consistent changes in both datasets, whereas an additional subset was unique to WT vs. Δ*rpoNp*::Ω*rpoNc*. The predominance of downregulated genes highlights chromosomal RpoNc as a global transcriptional activator under free-living nitrogen-fixing conditions and explains the panel organization in Figure 5A–C. Functional clustering identified eleven major RpoNc-regulated pathways, including nitrogen fixation-related processes (nitrogenase assembly, Fe–S cluster biogenesis, hydrogenase, heme biosynthesis, electron transfer, the GS–GOGAT pathway, glutamate metabolism, the urea cycle, and nitrate assimilation), carbon and amino acid metabolism, the TCA cycle, motility, cell-envelope biosynthesis, growth and cell division, ribosomal synthesis, infection/virulence, secretion systems, and cobalt/cobalamin metabolism (Figure 5).

### 2.4. RT-qPCR Validation of Differentially Expressed Genes (DEGs) in Key Metabolic and Cellular Pathways

To strengthen the interpretation of transcriptomic datasets in terms of DOA9 metabolism under free-living conditions, real-time quantitative PCR (RT-qPCR) was performed to validate the levels of expression of representative genes involved in carbon metabolism, amino acid metabolism, the TCA cycle, motility, cell-envelope biosynthesis, growth and cell division, ribosomal synthesis, infection/virulence, secretion systems, and cobalt/cobalamin metabolism. The RT-qPCR analysis was conducted on two groups of RpoN-regulated genes: (i) genes regulated by RpoNc (genes strongly downregulated in both WT vs. Δ*rpoNc* (RC) and WT vs. Δ*rpoNp::*Ω*rpoNc* (DB) comparisons), for which 40 representative genes were selected (Figures S3A,B) that were involved in several pathways, such as nitrogen activity, bacterial motility, cellular surface polysaccharides (CSPs), growth and cell division, the responsive system, and infection and virulence; and (ii) genes regulated by either RpoNc or RpoNp (genes downregulated exclusively in the WT vs. DB comparison, but still expressed in the WT vs. Δ*rpoNc* (RC) comparison), for which 28 genes were selected (Figure S3C) that were involved in pathways linked to motility, Fe–S cluster biogenesis, glutamate, CSP, growth and cell division, the responsive system, and quorum sensing. These validations confirmed the reliability of the transcriptome data and further supported the regulatory roles of RpoNc and RpoNp under free-living conditions. The RT-qPCR analysis showed the same expression trends as the RNA sequencing, confirming the reproducibility and dependability of the RNA sequencing results (Figure S3).

### 2.5. Global Metabolism Under the Control of RpoN in Free-Living DOA9

#### 2.5.1. RpoN Is Required for Free-Living Nitrogen Fixation and Nitrogen-Related Pathways Under Microaerobic Conditions

Nitrogen fixation is a highly energy-intensive process that requires coordinated activity across multiple metabolic pathways. Consistent with this, the transcriptome data revealed extensive transcriptional reprogramming in DOA9 under microaerobic N_2_-fixing conditions (Table S1 and Figure 3, Figure 4 and Figure 5 and Figure S4). Both the Δ*rpoNc* and Δ*rpoNp::ΩrpoNc* mutants exhibited strong downregulation of *nif* and *fix* gene clusters, consistent with their Fix^−^ phenotypes. Core chromosomal *nif* genes including *nifH*, *nifDK*, *nifE*, *nifN*, *nifX*, *nifV*, *nifB*, *nifZ*, *nifQ*, *nifW*, *rpoN*, and *nifU* were markedly repressed, along with plasmid-encoded *nifDK*. These genes encode structural and accessory components essential for catalytic nitrogenase assembly, FeMo-cofactor biosynthesis, and Fe–S cluster maturation, and their suppression indicates a severe impairment of nitrogenase formation in the absence of RpoN. Consistently, molybdenum transport genes (*modABC*, *modD*) required for FeMo-cofactor assembly were also strongly downregulated. A similar pattern was observed for *fix* genes including *fer1*, *fdx*, *fdxB*, *frxA*, and the *fixABCX* electron transfer complex, as well as regulatory genes (*fixJ*, *norV*, *fixBp*), highlighting the disruption of the microaerobic electron transfer network that supplies reducing power to nitrogenase.

In addition to nitrogenase assembly, RpoN disruption broadly affected multiple nitrogen-related metabolic pathways. Under wild type conditions, efficient N_2_ fixation depends on Fe–S cluster biogenesis, electron transport, hydrogen recycling, heme synthesis, and rapid NH_4_^+^ assimilation through the GS–GOGAT cycle. These interconnected systems were consistently suppressed in both mutants. Genes associated with urea degradation and transport (*ureA–D*, *urtA–D*, *braG*, *atzF*) were strongly downregulated, indicating a collapse of alternative ammonium-generating pathways. Genes responsible for nitrate–nitrite assimilation, including *nasA*, *nasD*, *nirA*, *nrtA*, *nrtB*, *fnt*, and the regulatory sensor kinase *ntrB,* were likewise repressed, demonstrating that RpoN is essential for maintaining nitrate–nitrite utilization under nitrogen-fixing conditions.

The GS–GOGAT cycle genes (*gltI*, *gltK*, *gltL*, *amtB*, *glnB*, *glnK*, *glnII*, and amidase *AF_1954*) were also reduced, reflecting impaired incorporation of NH_4_^+^ into glutamine and glutamate. Additional genes involved in amino acid turnover, including *thiO*, *atzE*, and those encoding several branched-chain amino acid-binding proteins, were downregulated, suggesting disrupted nitrogen–carbon flow following nitrogenase inactivation. Multiple peptide and sulfonate transporters (*dppB*, *dppD*, *dppF*, *ddpB*, *ddpC*, *oppF*, *y4tO*, *tauB*) were similarly suppressed, further indicating reduced nitrogen scavenging and recycling capacity.

Nitrogenase-supporting pathways were also broadly affected. Several *hya*-type hydrogenase genes (*hyaA*, *hyaB*, *hyaD*, *hyaC*, *alkJ*, *sthA*, *BRADOA9_v1_42127*) were strongly downregulated, suggesting reduced H_2_ recycling required to recover energy lost during nitrogenase turnover. Fe–S cluster biogenesis genes (*erpA*, *sufE*, *sufD*, *sufB*, *sufS*, *ycf64*, and related loci) were also suppressed, directly impairing cofactor assembly for nitrogenase. Genes encoding bacterial hemoglobins (*hbO*, *hemA*, *BRADOA9_v1_41488*) were downregulated, indicating weakened O_2_ buffering capacity, which, under normal conditions, protects nitrogenase from oxidative inactivation. Furthermore, genes associated with CO/CO_2_ redox regulation (*coxS*, *dmoA*, and related loci) were strongly repressed, suggesting impaired redox balance maintenance under microaerobic respiration.

#### 2.5.2. RpoN Regulates Cellular Adaptation Pathways

Beyond nitrogen fixation, RpoN also affected key cellular functions related to motility, surface structure formation, and nutrient acquisition (Table S1 and Figure 3, Figure 4 and Figure 5 and Figure S4). Several genes associated with type IV pilus regulation and cell-surface polysaccharides, including *pilZ*, *spsC*, *fecR*, *sraP*, *mraY*, and two additional loci (*BRADOA9_v1_41724*, *BRADOA9_v1_50948*), were consistently downregulated in the Δ*rpoN* mutants. The repression of *pilZ* and *spsC* suggests reduced pilus-mediated motility and adhesion, while downregulation of *mraY* indicates impaired peptidoglycan and surface polysaccharide biosynthesis.

Central carbon metabolic processes were also diminished, with reduced expression of *phaZ*, *mglA*, and three metabolic genes (*BRADOA9_v1_51781*, *BRADOA9_v1_41327*, *BRADOA9_v1_51377*). The repression of *phaZ* implies limited mobilization of carbon from PHA storage polymers, whereas reduced *mglA* expression points to decreased carbohydrate uptake, collectively indicating weakened carbon flow under nitrogen-fixing conditions.

Moreover, numerous permease transporter genes including BRADOA9_v1_51255, *BRADOA9_v1_51379*, *BRADOA9_v1_42218*, *BRADOA9_v1_51378*, *y4oQ*, *yadH*, and *yadG* were strongly downregulated. Their repression suggests reduced membrane transport capacity and restricted nutrient import, further constraining metabolic activity in the RpoN-deficient strains.

#### 2.5.3. RpoN Is Involved in Cell Division and Core Metabolic Pathways for Cell Growth

RpoN disruption also influenced cellular growth and division processes (Table S1 and Figure 3, Figure 4 and Figure 5 and Figure S4). Several genes associated with cell-cycle progression and morphogenesis including *bolA*, *prkC*, *alaS*, and multiple additional loci (*BRADOA9_v1_51544*, *BRADOA9_v1_42166*, *BRADOA9_v1_40434*, *atoh7*, *BRADOA9_v1_p0125*) were consistently downregulated in the Δ*rpoN* mutants. The repression of *bolA* (a global morphology regulator) and *prkC* (a serine/threonine kinase involved in cell-wall signaling) suggests impaired cell-shape control and cell division coordination, potentially contributing to the reduced growth capacity under microaerobic conditions. Genes involved in ribosomal assembly and protein synthesis were also markedly reduced, including *rplF*, *rpsQ*, *rpsE*, *rpsS*, *rplI*, *rplN*, *rplE*, *rluD*, and several ribosome-associated loci (*BRADOA9_v1_51382*, *BRADOA9_v1_50784*, *BRADOA9_v1_41972*, *BRADOA9_v1_p0365*). The widespread suppression of both the 30S and 50S subunit components highlights a global reduction in translational capacity, consistent with a general downshift in cellular growth activity in the RpoN-deficient strains. Several TCA-cycle-related genes, including *BRADOA9_v1_21692*, *BRADOA9_v1_43246*, *uctC*, *BRADOA9_v1_41906*, *BRADOA9_v1_42392*, and *BRADOA9_v1_40017*, were downregulated, suggesting reduced oxidative metabolism and energy generation. This repression aligns with the overall metabolic slowdown observed in the mutants. Additionally, genes associated with fatty-acid metabolism, BRADOA9_v1_50926 and *thi3*, were suppressed, indicating diminished lipid turnover and potential alterations in membrane biosynthesis. Together, these results demonstrate that RpoN contributes to sustaining cell division, protein synthesis, and core metabolic activity; thus, its loss leads to coordinated downregulation of growth-associated processes, reinforcing the central role of RpoN in maintaining cellular physiology under free-living nitrogen-fixing conditions.

#### 2.5.4. RpoN Influences Environment-Responsive and Interaction-Associated Systems Under Free-Living Microaerobic Conditions

Transcriptomic profiling revealed that a wide array of environment-responsive, stress-related, and interaction-associated genes were differentially regulated in the RpoN mutants, highlighting the broad adaptive role of RpoN under free-living microaerobic nitrogen-fixing conditions (Table S1 and Figure 3, Figure 4 and Figure 5 and Figure S4). Key regulators of iron and redox homeostasis, including *BRADOA9_v1_51509* (*irr*) and the oxidative stress-associated gene *BRADOA9_v1_50927*, were strongly downregulated, suggesting impaired control of heme biosynthesis, iron availability, and oxidative stress responses. Genes involved in nutrient and metal acquisition, such as *tonB*, were similarly repressed, indicating reduced siderophore-dependent uptake and micronutrient acquisition under minimal medium conditions.

Several regulatory and signaling genes including *nif11*, *traI*, *mtrA*, *yccV*, and *cynS* also showed reduced expression, reflecting broader disruption of environmental sensing pathways linked to nitrogen limitation, redox balance, and envelope-associated stress. Glutathione-related genes (*gsiA*, *gsiB*, *yddS*) were consistently downregulated, indicating diminished antioxidant capacity and compromised glutathione-mediated redox buffering. Additional stress-linked proteins, such as *BRADOA9_v1_50112* (signal peptide), *BRADOA9_v1_42225* (ABM-domain protein), *BRADOA9_v1_42100* (phenol hydroxylase), and *BRADOA9_v1_42168* (D-aminoacylase), were also affected, suggesting alterations in detoxification, membrane adaptation, and nutrient recycling.

Furthermore, several genes traditionally associated with infection- or virulence-like functions—such as *isochorismatase* genes (*BRADOA9_v1_41390*, *BRADOA9_v1_51380*), *amiE*, *pho*, an EF-hand calcium-binding protein (*BRADOA9_v1_50863*), a tetratricopeptide repeat protein (*BRADOA9_v1_51520*), *plK2* (serine/threonine kinase), *prkC* (cell-wall-responsive kinase), *araC* (environmental transcriptional regulator), a signal peptide gene (*BRADOA9_v1_20515*), and a plasmid-encoded hemolysin (*BRADOA9_v1_p0607*)—were also downregulated. Although not classic virulence factors in the pathogenic sense, these genes are typically involved in environmental interaction, stress adaptation, secretion, signaling, and early symbiotic or host-associated processes, indicating that RpoN contributes to maintaining the regulatory systems required for environmental communication and stress resilience.

Together, these transcriptional changes demonstrate that RpoN orchestrates a broad regulatory network extending beyond nitrogen fixation to include iron homeostasis, oxidative stress defense, redox-equilibrating mechanisms, environmental signal transduction, membrane adaptation, and interaction-associated pathways. These systems are crucial for DOA9 survival and function under minimal, microaerobic free-living nitrogen-fixing conditions.

#### 2.5.5. Unique DEGs in the Double RpoN Mutant Reveal Transcriptional Processes Potentially Influenced by the Plasmid-Encoded RpoNp

The gene set uniquely detected in the DOA9WT vs. Δ*rpoNp*::Ω*rpoNc* (double mutant of both *rpoNs*; DB) comparison represents transcriptional responses that appear only when both *rpoN* copies are absent. Because these DEGs do not occur in the Δ*rpoNc* single mutant, they likely reflect pathways specifically influenced by plasmid-encoded RpoNp or those requiring coordinated regulation by both RpoN sigma factors. Most genes exhibited moderate expression changes (log_2_FC ≈ −1 to −1.8), similar to the uniquely regulated DEGs in the Δ*rpoNc* dataset, indicating targeted regulatory disruption rather than global collapse.

A subset of DB-specific genes belonged to nitrogen fixation-related pathways. These included *nifH* and the plasmid-borne *rpoNp*, along with supporting components such as *BRADOA9_v1_42302* and *BRADOA9_v1_42305* (urea ABC-transporter substrate-binding proteins). Additional nitrogenase-supporting elements were detected, including *hyaD* (hydrogenase activity; *BRADOA9_v1_20912*), *ybiX* (sulfur dioxygenase), and *BRADOA9_v1_41342* (sulfur globule protein), suggesting impacts on Fe–S cluster biogenesis. Genes associated with the GS–GOGAT system (*BRADOA9_v1_40035*) and electron transfer processes (*trxC*/*BRADOA9_v1_50964*, *cycY*, and *BRADOA9_v1_41345*) further indicate localized impairment of nitrogen/redox metabolism unique to the double mutant.

Several genes involved in cellular adaptation and envelope-associated functions were uniquely downregulated, including *yjiB*, *rlpA*, *BRADOA9_v1_51082* (EGF-like glycoprotein), and *BRADOA9_v1_20901* (MFS-domain transporter). Two motility-related regulators, *BRADOA9_v1_50859* (CheY-like) and *BRADOA9_v1_50313* (*cheB*), were also detected, suggesting impaired chemotactic signaling. Genes related to amino acid and peptide transport (*oppD*, *braE*, *ybbK*) further point to altered nutrient acquisition strategies in the absence of both RpoNs.

Genes implicated in growth, DNA processing, and cell division were also uniquely represented. These included *pckA*, *serA*, *BRADOA9_v1_21519* (DNA topoisomerase IB), *smc1a*/*BRADOA9_v1_30257*, *BRADOA9_v1_43537* (terminase), *BRADOA9_v1_p0875* (transposase), *BRADOA9_v1_50952* (trigger factor), and *BRADOA9_v1_20612* (DUF4169). Additional DEGs mapped to environmental and stress-responsive systems, including *bfrD*, *norM*, *groL*, *hrp*, *BRADOA9_v1_20118* (adenylate cyclase), *BRADOA9_v1_40635*, *mscS, BRADOA9_v1_42478*, *selO*, *BRADOA9_v1_20681* (neuraminidase), and *BRADOA9_v1_41336* (PUF4 RNA-binding protein). Several stress- and quorum-sensing genes—*bepG*, *hspC(1)*, *htpG*, *BRADOA9_v1_20115* (adenylate cyclase), *htrA*, and *qheDH*—were uniquely detected in the DB dataset, highlighting specific regulatory functions associated with the loss of plasmid-encoded RpoNp.

Together, these findings demonstrate that the complete loss of both *rpoN* copies results in broader and more severe transcriptional defects than deletion of *rpoNc* alone. The unique DEGs detected in the Δ*rpoNc* comparison represent pathways specifically dependent on the chromosomal sigma factor, whereas the DB-specific DEGs reveal regulatory processes that require RpoNp or joint activity of both RpoN proteins. The diversity and functional breadth of DB-specific DEGs align with the pronounced physiological defects observed in the double mutant, affecting nitrogen fixation, redox balance, metabolism, stress adaptation, and multiple cellular pathways. These results highlight the complementary but nonredundant contributions of RpoNc and RpoNp in maintaining DOA9 fitness under microaerobic N_2_-fixing conditions. 

### 2.6. In Silico Identification of RpoN-Binding Sites 

**Figure 6:**
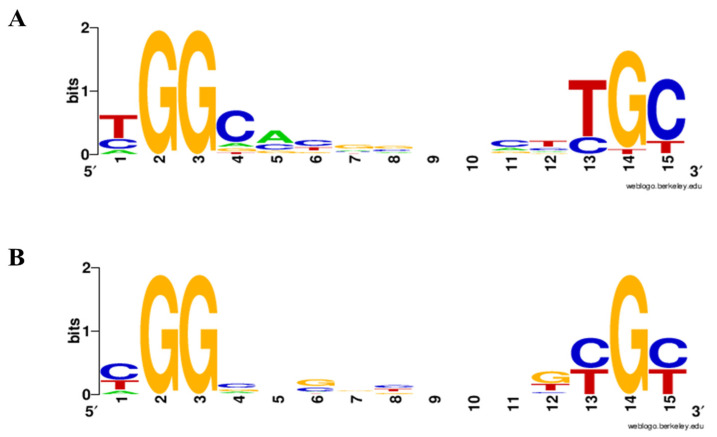
Consensus sequence logos of the RpoN box in *Bradyrhizobium* sp. DOA9. RpoN-specific consensus logos were generated from all detected RpoN-binding sequences using the WebLogo program. Logo (**A**) represents the consensus motif derived from RpoNc-binding sites, whereas logo (**B**) corresponds to the RpoNp-binding sites. Consensus motifs were constructed based on individual motifs listed in Table 1 and Table S2.

The differential expression and Venn diagram analyses described above indicated that RpoN influences gene expression in DOA9 through both direct promoter binding and indirect regulatory effects. To identify promoters that are likely under direct RpoN control, a genome-wide in silico search for RpoN-dependent promoter motifs was conducted. Following stringent filtering, a total of 68 high-confidence RpoNc-binding sites were identified across the DOA9 genome (Table 1; Figure S5). Of these, six motifs were located on the symbiotic megaplasmid, while the majority were distributed on the chromosome. The predicted RpoNc-binding sites were associated with functionally coherent gene groups, including 9 sites upstream of nitrogen fixation- and nitrogenase-associated genes; 13 sites linked to motility and cell-surface polysaccharide (CPS) biosynthesis; 23 sites associated with growth, cell division, and core metabolic pathways; and 20 sites upstream of genes involved in environment-responsive systems and cellular interaction processes. The close correspondence between the predicted RpoNc-binding motifs and transcriptome-defined DEGs supports the conclusion that these genes represent direct transcriptional targets of RpoNc during free-living nitrogen-fixing growth. A consensus sequence for RpoNc-binding sites was constructed by integrating motifs identified in this study with previously characterized RpoN-binding sites in the *nif* gene cluster [15]. The resulting motifs and sequence logos are shown in Figure 6 and summarized in Table 1, providing further support for direct promoter recognition by RpoNc [17].

In contrast, the in silico analysis of datasets derived from the WT vs. Δ*rpoNc* and WT vs. Δ*rpoNp::ΩrpoNc* comparisons identified only 22 putative RpoN-associated binding sites across the DOA9 genome (Table S2). These sites, which may be regulated by either RpoNc or RpoNp, were markedly fewer in number than the RpoNc-specific sites and were distributed across a limited subset of RpoN-responsive genes. A consensus sequence analysis revealed distinct nucleotide substitutions immediately upstream of the conserved σ^54^ signature dinucleotides. Specifically, the nucleotide preceding the −24 GG motif changed from thymine to cytosine (TGG → CGG), while the nucleotide preceding the −12 GC motif similarly shifted from thymine to cytosine (TGC → CGC) relative to the RpoNc-specific consensus (Figure 6). These motif differences indicate altered promoter architecture and are consistent with differential promoter recognition properties associated with RpoN paralogs.

Together, these results demonstrate that RpoN-dependent transcriptional regulation in DOA9 involves widespread direct promoter binding by the dominant chromosomal RpoNc, accompanied by a smaller and more restricted set of RpoN-associated binding sites with distinct motif features. This architecture supports broad RpoNc-mediated control of free-living nitrogen fixation and cellular physiology, while suggesting a more specialized or context-dependent contribution of the second RpoN homolog.

### 2.7. EMSA Validation of RpoN–Promoter Interaction with Both RpoNc and RpoNp

To determine whether the two RpoN sigma factors of DOA9 (RpoNc and RpoNp) directly recognize the promoter regions of target genes, Electrophoretic Mobility Shift Assays (EMSAs) were performed using purified recombinant RpoNc and RpoNp proteins. Protein expression constructs were generated using the pET22(–) vector, and soluble RpoN proteins obtained after cell lysis were subsequently purified and used for DNA-binding assays (Figure S6A–D).

**Figure 7:**
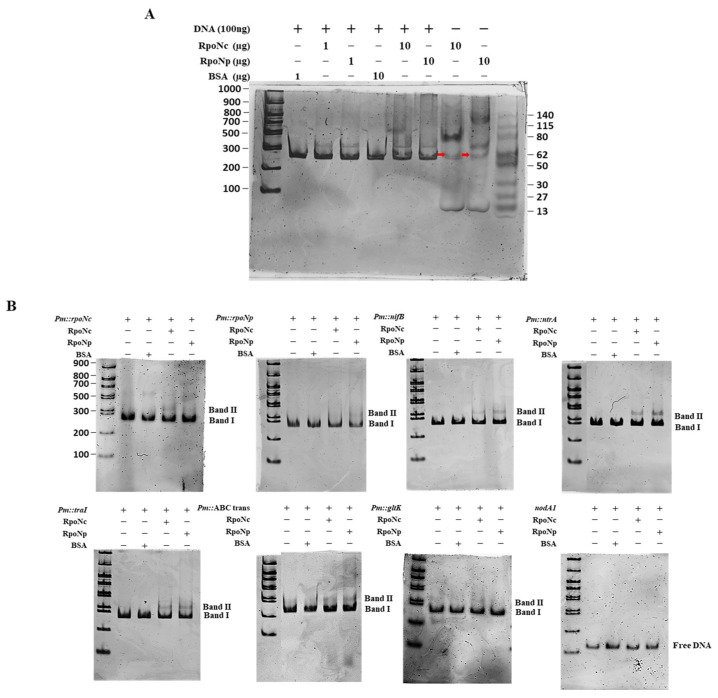
Electrophoretic Mobility Shift Assay (EMSA) analysis of RpoN binding to approximately 300 bp upstream sequences (promoter; *Pm*) of selected target genes. Screening reactions were performed with varying concentrations of RpoN protein and the upstream regions of the target DNA (exp; *Pm::hyaAc*), as indicated in the top panel. The gels were sequentially stained with RedSafe (first) and Coomassie blue (second) for visualization (**A**). The gel-shift results illustrate different *Pm* regions of the target genes, showing protein-bound DNA fragments (Band II) and free DNA fragments (Band I), comparable to the negative control (*nodA1*) (**B**). Images were captured with a 15-s exposure time. Red arrow indicates the protein band of RpoNc (left) and RpoNp (right), respectively.

Promoter fragments of approximately 300 bp containing predicted RpoN-binding sites were amplified based on the transcriptomic and motif prediction data. The selected promoter regions included *Pm::hyaAc*, *Pm::rpoNc*, *Pm::rpoNp*, *Pm::nifB*, *Pm::ntrA*, *Pm::traI*, *Pm::ABC-transporter*, and *Pm::gltK*, while *nodA1*, which lacks a σ^54^-binding motif, served as a negative control (Figure S7). To assess the protein–DNA interactions, EMSAs were performed using two protein concentrations (1 µg and 10 µg), both of which yielded comparable binding patterns for all of the promoters tested, indicating that binding efficiency was not markedly affected by protein abundance within this range. DNA–protein complexes were visualized using a two-step gel staining procedure: Redsafe DNA stain to detect DNA bands followed by Coomassie blue staining to visualize protein-containing complexes. Images of the results of both stains were merged to clearly identify the shifted complexes. Clear gel-shift signals (Band II) were observed for all of the tested promoter fragments when incubated with either RpoNc or RpoNp, whereas the unbound DNA migrated as Band I. No shifted bands were detected for the negative control (*nodA1*), confirming the specificity of the binding. The use of Coomassie blue staining further enabled visualization of the RpoN proteins in the control lanes, with distinct bands corresponding to purified RpoNc (60.57 kDa) and RpoNp (61.86 kDa), as shown in Figure 7A. Both RpoN proteins showed similar binding activity with most promoters; however, a faint shifted band was detected for *Pm::gltK* only with the RpoNc reaction, suggesting weaker or transient interaction with this promoter relative to the other targets. Overall, these results demonstrate that both RpoNc and RpoNp are capable of directly binding σ^54^-dependent promoters identified from transcriptomic analyses, supporting their roles in transcriptional regulation under free-living nitrogen-fixing conditions.

### 2.8. Comprehensive Regulatory Model of RpoN-Dependent Control of Free-Living Conditions and Nitrogen Fixation in Bradyrhizobium sp. DOA9

**Figure 8:**
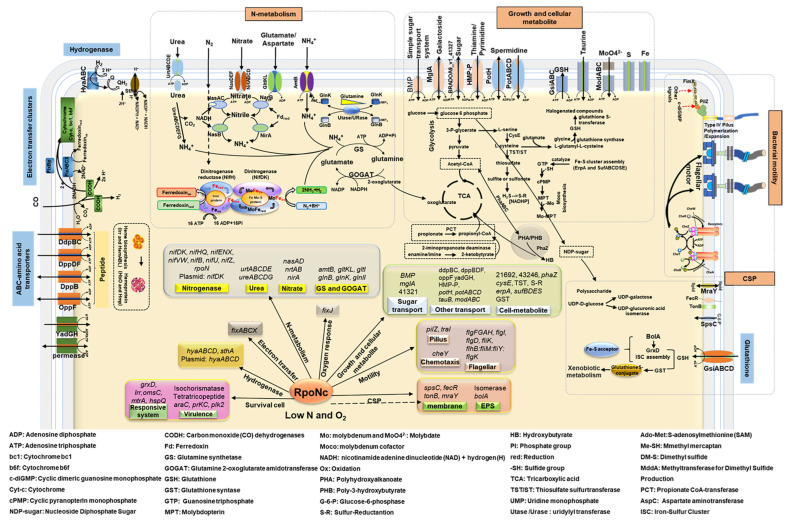
A proposed schematic model of RpoN regulatory networks in free-living *Bradyrhizobium* sp. DOA9 under microaerobic conditions.

Transcriptomic profiling supported by RT-qPCR validation and functional categorization collectively indicated that RpoN functions as a central regulatory hub coordinating nitrogen fixation, core metabolism, cellular growth, and environmental adaptation in DOA9 under free-living microaerobic conditions (Figure 8). Among the two RpoN homologs, chromosomally encoded RpoNc emerges as the dominant σ^54^ factor, exerting primary control over the nitrogenase gene network and the associated physiological systems required to sustain nitrogen fixation outside the symbiotic context.

The schematic model summarizes the RpoN-dependent regulatory networks inferred from transcriptomic analysis and validated by RT-qPCR, highlighting the central role of RpoN in coordinating nitrogen metabolism, cellular physiology, and environmental adaptation during free-living conditions. Regulation is initiated from nitrogen fixation related processes, where RpoN, predominantly the chromosomally encoded RpoNc, controls the expression of *nif* and *fix* gene clusters required for nitrogenase assembly, hydrogenase activity, electron transfer, molybdenum transport, heme biosynthesis, and microaerobic respiration. Downstream of nitrogen fixation, RpoN integrates nitrogen assimilation and recycling pathways, including urea utilization, nitrate assimilation, and the GS–GOGAT cycle, thereby linking fixed nitrogen to amino-acid metabolism and maintaining nitrogen–carbon balance. RpoN-dependent control further extends to oxygen-responsive systems that support energy generation and redox balance under microaerobic conditions.

Beyond nitrogen metabolism, RpoN influences core physiological processes related to growth and cell division, as well as motility and surface architecture. Reduced expression of genes involved in flagellar assembly, type IV pili regulation, and cellular surface polysaccharide biosynthesis reflects impaired motility, attachment, and environmental interaction. In parallel, repression of pathways associated with carbon utilization and storage suggests constrained metabolic flexibility. RpoN also modulates stress response and environmental adaptation mechanisms, including glutathione-based oxidative stress defense, iron and redox regulation, and multiple transport systems for nutrients and signaling molecules. Collectively, the schematic illustrates how RpoN functions as a global regulatory hub that synchronizes nitrogen fixation with metabolic capacity, cellular growth, motility, surface structure formation, and stress responses, enabling free-living *Bradyrhizobium* sp. DOA9 to adapt to microaerobic environments outside the symbiotic context.

Consistent with this role, strong repression of the *nif* and *fix* gene clusters in the Δ*rpoNc* and double mutants highlights the dependence of nitrogenase assembly, hydrogenase activity, molybdenum transport, heme biosynthesis, and microaerobic electron transfer on RpoNc-mediated transcription. In parallel, the coordinated downregulation of urea and nitrate assimilation pathways, the GS–GOGAT cycle, amino acid turnover routes, and multiple peptide and sulfonate transport systems indicates that RpoNc links nitrogen fixation to nitrogen scavenging and the maintenance of the nitrogen–carbon balance during free-living conditions.

Beyond nitrogen metabolism, RpoN-dependent regulation extends to cellular structures and adaptive traits essential for environmental fitness. Reduced expression of flagellar and motility-associated genes (*flgF*, *flgI*, *flhB–fliMY*), surface and polysaccharide biosynthesis genes (*spsC*, *mraY*, CPS-related enzymes), and type IV pilus regulators (including *pilZ*) reflects impaired motility and weakened surface-structure formation, which may affect attachment and environmental interactions. Concomitant repression of carbon metabolic genes such as *phaZ*, *mglA*, and related catabolic enzymes suggests diminished carbohydrate utilization and limited mobilization of polyhydroxyalkanoate (PHA) reserves.

Transport capacity and metabolic flexibility were also broadly constrained in the absence of functional RpoN. Multiple transporter systems, including sugar permeases, nickel- and peptide-binding proteins, and spermidine or polyamine transporters (*potH*, *potC*, *ydcT*), were collectively downregulated, indicating reduced nutrient uptake and membrane transport activity. In addition, RpoN influences cellular growth and bioenergetic capacity, as reflected by the decreased expression of genes involved in cell-shape maintenance and division (*bolA* paralogs, *prkC*, *alaS*), along with the widespread repression of ribosomal proteins and assembly factors from both the 30S and 50S subunits. The suppression of tricarboxylic acid cycle components and fatty-acid metabolism genes further suggests reduced energy production and membrane biosynthesis under RpoN-deficient conditions.

RpoN also integrates environment-responsive and interaction-associated systems critical for survival in microaerobic minimal media. The downregulation of iron- and redox-related genes (*irr* and oxidative stress-associated proteins), nutrient acquisition systems (*tonB*), signaling and regulatory components (*nif11*, *traI*, *mtrA*, *cynS*), and glutathione metabolism genes (*gsiA*, *gsiB*, *yddS*) indicates compromised redox homeostasis, oxidative defense, and environmental sensing. Notably, several genes linked to secretion, surface communication, and early symbiotic competence including isochorismatases, EF-hand proteins, TPR-repeat proteins, AraC-family regulators, and plasmid-encoded hemolysin were also downregulated, suggesting that RpoN contributes to maintaining interaction readiness even during free-living conditions.

## 3. Discussion

This study demonstrates that DOA9 employs two σ^54^ (RpoN) homologs with complementary yet nonredundant functions during free-living conditions under microaerobic nitrogen-fixing conditions. *Bradyrhizobium* species display remarkable lifestyle versatility, growing under aerobic, microaerobic, and symbiotic conditions within legume nodules; accordingly, global transcriptional responses are strongly environment-dependent. Here, transcriptomic analyses were conducted under a defined free-living microaerobic condition designed to mimic the low-oxygen environment required for in vitro nitrogenase activity. Although this condition differs from fully aerobic growth and plant symbiosis, it provides a controlled framework for evaluating nitrogenase function and growth phenotypes of the wild type and *rpoN* mutants. Therefore, the regulatory roles described here should be interpreted as specific to free-living nitrogen-fixing conditions, and future studies under aerobic and symbiotic environments will be necessary to determine the broader environmental scope of RpoN regulation.

While σ^54^ is classically linked to nitrogen fixation through the activation of *nif* genes, our comparative transcriptomic and phenotypic analyses demonstrate a broader regulatory landscape in DOA9, in which RpoNc acts as the principal global regulator and RpoNp provides additional regulatory capacity that becomes evident when both σ^54^ functions are removed. This functional partitioning aligns with the notion that the duplication and divergence of σ^54^ systems can support regulatory specialization across environmental and lifestyle transitions in rhizobia [12,18,19].

Our phylogenetic analyses support this interpretation (Figure 1), showing that RpoNc groups with RpoN proteins from symbiosis-associated bradyrhizobia, whereas RpoNp forms a distinct clade, consistent with functional divergence. This separation is further supported by phylogenies based on housekeeping genes and core nitrogen-fixation proteins (e.g., 16S rRNA and NifHDK), which place DOA9 firmly within the symbiotic *Bradyrhizobium* lineage while highlighting the atypical evolutionary trajectory of the plasmid-encoded RpoNp. The distribution of the two paralogs across replicons reinforces this view: the chromosomal *rpoNc* is conserved and broadly distributed, indicating long-term integration into the core regulatory network, whereas *rpoNp* resides on the symbiotic plasmid and shows a patchier evolutionary distribution, consistent with acquisition and specialization through horizontal gene transfer. Notably, RpoN sequences from free-living or non-nodulating strains cluster with the “copy2” clade in several lineages, suggesting that the RpoNp-like group may be evolutionarily tuned toward free-living adaptation rather than canonical symbiotic control [19,20,21]. Together, the phylogenetic, replicon, and functional evidence supports a σ^54^ “generalist–specialist” architecture in DOA9 [19,22], in which chromosomal RpoNc governs core transcriptional programs required for growth and nitrogen fixation under microaerobic conditions, while plasmid-encoded RpoNp contributes additional adaptive and regulatory capacity.

Transcriptomic repression (Figure 3) extended beyond the core *nif* genes to encompass molybdenum transport (*modABC*), Fe–S cluster assembly (*suf*/*erpA*), and electron transfer systems (*fixABCX*), mirroring regulatory architectures reported in *B. japonicum* and *B. diazoefficiens* during nitrogen-fixing growth [23,24,25]. Similar σ^54^-dependent coordination of nitrogenase-supporting pathways has also been described in other diazotrophs, indicating a conserved strategy for integrating cofactor biosynthesis, redox balance, and oxygen protection [26,27,28]. Efficient nitrogen fixation requires tight coupling between nitrogenase activity and downstream nitrogen assimilation. In DOA9, σ^54^ disruption suppressed nitrate–nitrite assimilation (*nas/nir/nrt*), urea utilization, and GS–GOGAT pathway genes (Figure 4 and Figure 5), consistent with findings in *Bradyrhizobium* spp. showing that σ^54^ integrates nitrogen fixation with broader nitrogen metabolism [29,30,31]. Similar regulatory linkages have been observed in *B. diazoefficiens*, where nitrogenase activity, ammonium assimilation, and nitrogen-responsive transport systems are transcriptionally coordinated [19]. Although specific roles in peptide or sulfonate transport have not been previously described for σ^54^ in rhizobia, the repression of these transporters observed here is consistent with broader σ^54^-linked regulation of nutrient utilization. Prior transcriptomic studies have shown that σ^54^ impacts nitrogen fixation and associated transport processes in rhizobia, including C4-dicarboxylate transport [32]. Additionally, σ^54^-dependent regulation of carbon and nitrogen metabolic pathways has been documented in diverse bacteria, supporting the observed downregulation of TCA cycle genes [22].

σ^54^-dependent regulation in DOA9 extended to motility-, chemotaxis-, and cell-envelope-associated pathways (Figure 4 and Figure 5). Similar roles for σ^54^ in regulating motility and surface structures have been reported in *Rhizobium* and *Sinorhizobium* species, in which these traits contribute to environmental adaptation and host interactions [33,34,35]. The downregulation of polysaccharide and envelope biosynthesis genes in the σ^54^-deficient strains was accompanied by altered colony morphology and increased stress sensitivity in DOA9, suggesting that RpoN contributes to maintaining cell-surface integrity and stress resilience under free-living conditions. 

Although RpoNc dominated the global transcriptional response, the presence of DB-specific DEGs indicates additional regulatory processes potentially influenced by plasmid-encoded RpoNp or joint σ^54^ activity (Figure 4 and Figure 5). The generally moderate expression changes and enrichment for stress response, transport, and signaling functions are consistent with secondary or accessory σ^54^ regulons described in rhizobia with multipartite genomes, where plasmid-encoded regulators contribute to regulatory robustness and environmental flexibility rather than replacing chromosomal core functions [18,19,36]. In *Bradyrhizobium* spp., such division of regulatory labor has been proposed to support adaptive responses under fluctuating environmental conditions [1,18,37]. 

Genome-wide motif analysis identified canonical −24/−12 σ^54^-binding sequences upstream of many DEGs (Table 1 and Table S2 and Figure 6), consistent with σ^54^ promoter usage reported for nitrogen-fixation and metabolic genes in *Bradyrhizobium* [23,24,25,38]. EMSA further demonstrated that both RpoNc and RpoNp directly bind these σ^54^-dependent promoters, supporting overlapping DNA recognition (Figure 7). Notably, a faint shifted band was observed for the *Pm::gltK* promoter only in the presence of RpoNc, suggesting weaker or more transient binding compared with other targets. This may reflect lower affinity caused by subtle deviations from the σ^54^ consensus sequence or context-dependent promoter architecture. Nevertheless, productive σ^54^-dependent transcription requires activation by bacterial enhancer-binding proteins (bEBPs); therefore, differences in bEBP availability, specificity, or signal responsiveness likely underlie functional differentiation between the two σ^54^ factors despite shared promoter recognition, as established in other σ^54^-regulated systems [6,39,40]. In silico motif analyses and genome-wide studies of σ^54^ regulons support the existence of broad σ^54^ control over diverse pathways beyond classical nitrogen metabolism. Genome-wide identification of σ^54^ targets has been reported in *E. coli* using ChIP and transcriptomics, revealing dozens of σ^54^ promoters and demonstrating expansive regulon structure [41]. Comparative reconstruction across multiple genomes further indicates that σ^54^ regulons vary widely in size and gene content, consistent with accessory and core σ^54^ functions [42]. However, EMSA only demonstrated promoter binding and does not confirm transcriptional activation. Therefore, the involvement of bEBPs in σ^54^-dependent transcription and their roles in activating target gene expression remain to be investigated in future studies.

Taken together, our data support a regulatory model in which chromosomally encoded RpoNc acts as the primary σ^54^ factor coordinating nitrogen fixation, nitrogen assimilation, and growth-associated processes during free-living microaerobic growth (Figure 8). This role is consistent with σ^54^-dependent regulatory architectures described in other *Bradyrhizobium* species, in which σ^54^ integrates nitrogenase expression with supporting metabolic and physiological pathways required for nitrogen-fixing activity under oxygen-limited conditions [24,25,43]. In contrast, plasmid-encoded RpoNp provides complementary regulatory input that becomes evident only when σ^54^ control is globally compromised, contributing to the regulation of stress response, transport, and interaction-associated pathways rather than core nitrogen fixation functions.

Such a dual-σ^54^ organization likely enhances transcriptional flexibility and regulatory robustness in DOA9, a strain characterized by duplicated *rpoN* and *nifA* genes distributed across the chromosome and symbiotic plasmid. Similar regulatory partitioning has been proposed for rhizobia with multipartite genomes, in which accessory regulators fine-tune environmental adaptation without replacing chromosomal master regulators [18,36,37]. Together, these findings highlight σ^54^ diversification as a key evolutionary strategy that enables free-living nitrogen-fixing *Bradyrhizobium* to integrate environmental sensing with core metabolic programs, thereby optimizing physiological fitness under microaerobic conditions.

## 4. Materials and Methods

### 4.1. Bacterial Strains and Culture Conditions

Wild type *Bradyrhizobium* sp. DOA9 (DOA9WT) and all *rpoN* mutant strains including ∆*rpoNc*, ∆*rpoNp*, and ∆*rpoNp*::Ω*rpoNc* were obtained from the Applied Soil Microorganism Laboratory, School of Biotechnology, Suranaree University of Technology, Thailand [15]. The bacterial strains were cultured in yeast extract–mannitol (YEM) medium, prepared by dissolving 10 g (Sigma-Aldrich, St. Louis, MO, USA), 0.5 g KH_2_PO_4_ (Sigma-Aldrich, St. Louis, MO, USA), 0.5 g MgSO_4_·7H_2_O (Sigma-Aldrich, St. Louis, MO, USA), 0.5 g NaCl (Sigma-Aldrich, St. Louis, MO, USA), and 1 g yeast extract (BD Difco, Franklin Lakes, NJ, USA per liter of distilled water, with the pH adjusted to 6.8. To activate the strains, DOA9WT and its derivatives were streaked onto yeast extract–mannitol (YEM) agar plates (pH 6.8) and incubated at 30 °C for 5 days. A single colony from each strain was then transferred into YEM broth (pH 6.8) and incubated at 30 °C with shaking at 150 rpm for 5 days in a MaxQ 6000 Incubated/Refrigerated Stackable Shaker (SHKE6000-8CE; Thermo Scientific, Waltham, MA, USA). To optimize culturing conditions, appropriate antibiotics were added to the medium at the following concentrations: 300 µg/mL kanamycin (Sigma-Aldrich, St. Louis, MO, USA), 300 µg/mL streptomycin (Sigma-Aldrich, St. Louis, MO, USA), 20 µg/mL nalidixic acid (Sigma-Aldrich, St. Louis, MO, USA), and 20 µg/mL cefotaxime (Sigma-Aldrich, St. Louis, MO, USA). After incubation, the cells were harvested and washed twice with BNM-B medium without succinate (BNM-B*; pH 6.8) by centrifugation at 4000× *g* at 4 °C for 10 min. The washed cells were then resuspended in BNM-B* medium, and the cell concentration was measured at an optical density (OD) of 600 nm. The OD_600_ was adjusted to 15 [15].

### 4.2. RNA Preparation and Sequencing for Gene Expression in DOA9WT and Mutant Strains Under Free-Living Conditions

To determine gene expression in *Bradyrhizobium* sp. strain DOA9 (DOA9WT) and its derivative mutants under free-living conditions, 1% (*v/v*) prepared cell suspensions of each strain were inoculated into 150 mL glass bottles containing 50 mL of BNM-B broth and incubated statically at 30 °C for 7 days [14]. Cells were harvested through centrifugation at 4000× *g* at 4 °C for 10 min, and total RNA was extracted using the Flavogen RNA Purify Kit (Qiagen, Hilden, Germany) followed by DNase I treatment (Thermo Fisher Scientific, Waltham, MA, USA) to remove genomic DNA contamination. RNA samples were submitted to GENEWIZ Biotechnology Co., Ltd. (China) for RNA sequencing. Libraries were prepared and pooled based on effective concentrations and the required sequencing data volume, and sequencing was performed on an Illumina platform. Each condition was analyzed in biological triplicates, resulting in nine libraries, with 21 to 26 million sequences obtained per sample and 91.1% to 98.6% of reads mapped to the genome of *Bradyrhizobium* sp. strain DOA9 (WGS; genome data sourced from the NCBI database). Sequencing data were deposited in the Sequence Read Archive (SRA) under accession number GSE108744 (SRA: SRP128034). Differentially expressed genes (DEGs) were annotated using Gene Ontology (GO) and KEGG pathway analyses and integrated into a metabolic network to visualize the regulation mediated by the two RpoN proteins in *Bradyrhizobium* sp. strain DOA9 under free-living conditions (refer to Supplementary Methods S1 for additional methods used for RNA-Seq data analysis).

### 4.3. RT-qPCR Analysis for Gene Expression in DOA9WT and Mutant Strains Under Free-Living Conditions

Reverse transcription quantitative PCR (RT-qPCR) was carried out to determine the gene expression in DOA9WT and *rpoN* mutants under free-living conditions. Purified RNA samples were reverse-transcribed into complementary DNA (cDNA) using the iScript™ Reverse Transcription Supermix (Bio Rad Laboratories, Hercules, CA, USA), and reactions were performed with THUNDERBIRD^®^ SYBR^®^ qPCR Mix (TOYOBO Co., Ltd., Osaka, Japan) using specific primers (Table S3). Relative expression levels of target genes were normalized to the housekeeping gene 16S rRNA and analyzed using QuantStudio™ Design & Analysis Software (Applied Biosystems, Thermo Fisher Scientific, Waltham, MA, USA) [44].

### 4.4. In Silico Analysis of RpoN-Binding Regions in Bradyrhizobium sp. DOA9 Genome

Sigma factor RpoN (σ^54^) typically recognizes the conserved −24/−12 promoter consensus sequence 5′-YTGGCACGrNNNTTGCW-3′, in which the −24 “GG” and −12 “GC” dinucleotides represent key signature elements required for σ^54^-dependent transcription initiation. Based on sequence variability reported in other nitrogen-fixing bacteria [12,13,14], additional nucleotide variants at the −12 region (TTT, TTA, CGT, CGC, CTT, and CTA) were included to increase the sensitivity of motif detection in the DOA9 genome.

Putative RpoN-binding motifs were searched for within −500 bp upstream of annotated start codons using the Infectio motif search tool, followed by motif alignment and verification with MEGA11. Candidate sites were subsequently cross-referenced with transcriptome-defined RpoN-responsive genes (z-score ≥ 2.0), thereby restricting the analysis to promoters associated with differentially expressed genes. The initial genome-wide scan detected more than 2000 putative RpoN-like motifs (data not shown). These candidates were further filtered based on (i) overlap with DEGs, (ii) genomic context evaluated using the Microscope annotation platform, and (iii) precise motif conservation confirmed by MEGA11 alignment.

### 4.5. Validation of RpoN-Regulated Gene Interactions Using Electrophoretic Mobility Shift Assay (EMSA)

To validate the RpoN-regulated genes identified from the transcriptomic data, we performed an Electrophoretic Mobility Shift Assay (EMSA) to investigate the DNA-binding interaction of RpoNc and RpoNp with the upstream regulatory regions of target genes. The *rpoN* gene was cloned into the pET22(+) vector with an N-terminal 6 × His tag and expressed in *Escherichia coli* BL21(+) cells. The recombinant RpoN-His6 protein was purified using affinity chromatography (Biocomma Frits columns, volume 1 mL to 300 mL, pore size 50 μm), resuspended in elution buffer containing 20 mM Tris, 250 mM NaCl, and 200 mM imidazole, and stored at −80 °C after concentration measurement. For DNA fragment preparation, approximately 300 bp of the upstream regulatory regions of target genes was amplified via PCR using *Taq* polymerase (*DeamTaq*, Thermo Fisher Scientific, Waltham, MA, USA) with gene-specific primers (Table S2). The EMSA reaction was conducted in a 20 µL mixture containing 10 µg/mL purified RpoN-His6 protein and 100 ng/mL of the selected gene promoter DNA fragment in binding buffer, incubated at room temperature in the dark for 15 min [45]. If the protein binds to the nucleic acid, a protein–DNA complex forms, which migrates slower during polyacrylamide gel electrophoresis than unbound probes. A “shifted” band on the gel indicates binding interaction between the protein and the DNA probe [46]. After incubation, Pierce™ Coomassie Brilliant Blue dye (Thermo Fisher Scientific, Waltham, MA, USA) was added for visualization, and the samples were subjected to electrophoresis in a native polyacrylamide gel at 10 V/cm, in accordance with the protocol by Hsieh et al. [47]. The gel was scanned directly in glass plates using the Bio-Rad ChemiDoc Touch Imaging System (Bio-Rad Laboratories, Hercules, CA, USA) to detect protein–DNA interactions.

### 4.6. Statistical Analysis

In this study, phylogenetic trees were constructed using bioinformatic analyses in MEGA version 11 [48]. Statistical analyses were performed using SPSS Statistics version 26.0 (IBM, Armonk, NY, USA). Data are presented as the mean ± standard error (SE). Significant differences were assessed using one-way analysis of variance (ANOVA) followed by Duncan’s multiple range test, and general linear model (GLM) ANOVA followed by Tukey’s post hoc test. A *p* value of <0.05 was considered statistically significant [49,50]. Mutant construction and sequence analyses were conducted using SnapGene, the online Genoscope platform, and AmplifX [51,52,53].

## 5. Conclusions

This study demonstrates that the two RpoN proteins of *Bradyrhizobium* sp. DOA9 play distinct yet partially overlapping regulatory roles, with the chromosomal RpoNc functioning as the primary σ^54^ factor required for free-living conditions and efficient nitrogen fixation. Data presented in this research establish RpoNc as a global regulatory hub that integrates nitrogen fixation with nitrogen assimilation, central metabolism, cellular growth, and stress adaptation under free-living microaerobic conditions (Figure 8). The coordinated repression of the *nif* and *fix* genes, nitrogen assimilation pathways, transport systems, ribosomal components, energy metabolism, and environmental sensing modules in the RpoNc-deficient strains highlights the dependence of free-living nitrogen fixation on a tightly coupled RpoN-centered regulatory network. In this framework, RpoNp appears to provide accessory or condition-dependent regulation, supporting regulatory diversification in DOA9. Together, this dual-RpoN architecture reveals a hierarchical σ^54^ regulatory system that underpins metabolic integration and physiological fitness in free-living nitrogen-fixing *Bradyrhizobium*.

## Figures and Tables

**Table 1:** The specific binding site of RpoN, located upstream of a single predicted gene and potentially regulated by the RpoNc protein, is presented alongside gene information classified by metabolic pathway.

Metabolite/Gene	Description	RpoN-Binding Site 5′ >3′	Position	Sequence ID (Genoscope)	Sequence ID (NCBI)
*fixJ*	Transcriptional regulatory protein FixJ	TGGCGTGAATGCCGT	−76	BRADOA9_v1_40695	BDOA9_RS11995
*fixA*	Electron transport, Nitrogen fixation, FixA	TGGTACGACACTTGC	−63	BRADOA9_v1_51505	BDOA9_RS30700
*modA*	Molybdate-binding protein ModA	CGGCACGCCGATTGC	−72	BRADOA9_v1_51497	BDOA9_RS30665
*cheY*	Chemotaxis protein CheY	CGGAACGGAAATTGC	−38	BRADOA9_v1_51515	BDOA9_RS30735
*flgF*	Flagellar basal-body rod protein FlgF	TGGCACGGCTTTCGC	−112	BRADOA9_v1_51018	BDOA9_RS28660
*flgI*	Flagellar P-ring protein 1	AGGGCGAACGAGTGC	−168	BRADOA9_v1_51030	BDOA9_RS28710
*flhB*, *fliM*, and *fliY*	Flagellar basal body protein	AGGCCTTGCGACTGT	−59	BRADOA9_v1_51050	BDOA9_RS28785
*urtA-B-C*	Urea ABC transporter, Urea binding protein	TGGCATGGTTCTTGC	−77	BRADOA9_v1_40426	BDOA9_RS10850
*glnII*	Glutamine synthetase	TGGCACGCGAAATGC	−93	BRADOA9_v1_42831	BDOA9_RS20900
*amtB*	Ammonia/ammonium transporter	AGGGGTCCCGGCTGT	−88	BRADOA9_v1_21477	BDOA9_RS06210
*glnB*	Nitrogen regulatory protein PII-1	TGGCATAGACCCTGC	−174	BRADOA9_v1_50070	BDOA9_RS24700
*BRADOA9_v1_42224*	Putative amidase AF_1954	CGGTATACCGCCTGC	−188	BRADOA9_v1_42224	BDOA9_RS18415
*gltK*	Glutamate/aspartate ABC transporter membrane subunit GltK	TGGCCGGACATTTGC	−47	BRADOA9_v1_40129	BDOA9_RS09625
*BRADOA9_v1_42127*	Uptake hydrogenase small subunit precursor	TGGCACCGGCCATGC	−46	BRADOA9_v1_42127	BDOA9_RS32350
*hyaA*	Hydrogenase 1 small subunit	TGGCCTGCTTCTTGC	−46	BRADOA9_v1_42129	BDOA9_RS18000
*hyaA*	Hydrogenase 1 small subunit	TGGCCCGCTTCTTGC	−45	BRADOA9_v1_p0690	BDOA9_RS35605
*sthA*	Putative soluble pyridine nucleotide transHydrogenase	TGGCCCGATTGCTGC	−42	BRADOA9_v1_21705	BDOA9_RS07165
*nrtA*	Nitrate/nitrite binding protein NrtA	CGGGGGTTCCCCCGT	−103	BRADOA9_v1_41484	BDOA9_RS15300
*fnt*	Nitrite transporter from formate/nitrite family	CGGCAGGCCGCCCGC	−74	BRADOA9_v1_41483	BDOA9_RS15295
*BRADOA9_v1_50923*	Nitrate/nitrite transport system substrate-binding protein	TGGCATGCTCCTTGC	−36	BRADOA9_v1_50923	BDOA9_RS28275
*BRADOA9_v1_43377*	NAD(P)H-dependent nitrite reductase catalytic subunit	TGGCCGCAGGAGCTT	−12	BRADOA9_v1_43377	BDOA9_RS23185
*BRADOA9_v1_43052*	The PilZ protein family	CGGTATCACTTGCTT	−57	BRADOA9_v1_43052	BDOA9_RS21810
*shc*	Squalene-hopene cyclase	AGGACATCCGCGCGT	−12	BRADOA9_v1_41724	BDOA9_RS16295
*spsC*	Spore coat polysaccaharide biosynthesis protein SpsC	CGGCTCTTAGGACGT	−55	BRADOA9_v1_51531	BDOA9_RS30800
*fecR*	domain-containing protein FecR	AGGACTTGGTAATGC	−58	BRADOA9_v1_20513	BDOA9_RS35355
*sraP*	Conserved protein of unknown function sraP	AGGCCTTGCGACTGT	−58	BRADOA9_v1_51048	BDOA9_RS28775
*rpsE*	30S ribosomal subunit protein S5	TGGAGAAGGCGATGC	−224	BRADOA9_v1_50507	BDOA9_RS26510
*rplF*	50S ribosomal subunit protein L6	CGGTGAAGAACCTGC	−133	BRADOA9_v1_50509	BDOA9_RS26520
*rplW*	50S ribosomal subunit protein L23	CGGCCTGACCAACGC	−169	BRADOA9_v1_50524	BDOA9_RS26585
*BRADOA9_v1_51382*	Cytidine/deoxycytidylate deaminase family protein	TGGCACGAAGCTTGC	−76	BRADOA9_v1_51382	BDOA9_RS30175
*BRADOA9_v1_50784*	CP_ATPgrasp_1 domain-containing protein	TGGCCCGGCCCTTGC	−113	BRADOA9_v1_50784	BDOA9_RS27680
*rplI*	50S ribosomal protein L9	CGGGCTTGCGGATGC	−115	BRADOA9_v1_42710	BDOA9_RS20395
*rluD*	Ribosomal large subunit pseudouridine synthase D	TGGCGGTGACATCGC	−184	BRADOA9_v1_20316	BDOA9_RS34655
*bolA*	Cell division protein BolA	CGGCATGGCTGACTT	−103	BRADOA9_v1_20940	BDOA9_RS30755
*BRADOA9_v1_42166*	Cytosine/purine/uracil/thiamine/allantoin Permease family protein	TGGCACGATTTATGC	−39	BRADOA9_v1_42166	BDOA9_RS18140
*hddc3*	HD domain-containing protein (Guanosine-3′,5′-bis(diphosphate) 3′-pyrophosphohydrolase MESH1)	TGGCATGGTTCTTGC		BRADOA9_v1_40434	BDOA9_RS10890
*atoh* *7*	Transcription factor Atoh7	CGGCAACAACGCCTA	−33	BRADOA9_v1_41679	BDOA9_RS27685
*prkC*	Protein kinase Serine/threonine-protein kinase	CGGCAGGCCGCCCGC	−74	BRADOA9_v1_41482	BDOA9_RS15290
*BRADOA9_v1_41937*	ATP-grasp domain-containing protein	TGGCATACCACATGC	−247	BRADOA9_v1_41937	BDOA9_RS17170
*BRADOA9_v1_50927*	OsmC-like protein	TGGCACGCTCCATGC	−58	BRADOA9_v1_50927	BDOA9_RS28295
*BRADOA9_v1_41019*	Glutathione S-transferase	TGGCACGTCGCTTGC	−69	BRADOA9_v1_41019	BDOA9_RS13350
*BRADOA9_v1_50112*	Secreted protein or Signal peptide protein	TGGCACTCCGCTTGC	−41	BRADOA9_v1_50112	BDOA9_RS24880
*BRADOA9_v1_42225*	ABM domain-containing protein	CGGCCCGGTATGCTT	−40	BRADOA9_v1_42225	BDOA9_RS18420
*BRADOA9_v1_42100*	2-polyprenyl-6-methoxyphenol hydroxylase	AGGGAATTGCACTGC	−30	BRADOA9_v1_42100	BDOA9_RS17885
*BRADOA9_v1_42168*	D-aminoacylase	TGGCACGAAGCTTGC	−102	BRADOA9_v1_42168	BDOA9_RS18150
*BRADOA9_v1_p0683*	Nicel/Cobalt-specific TonB-dependent outer membrane receptor	TGGCCTGGCTCTTGC	−47	BRADOA9_v1_p0683	BDOA9_RS35575
*traI*	Putative acyl-homoserine-lactone synthase	CGGGACCGCGCCTGC	−54	BRADOA9_v1_p0112	BDOA9_RS33765
*nif* *11*	Nif11 domain-containing protein	TGGCACGCTCCTTGC	−76	BRADOA9_v1_p0609	BDOA9_RS35350
*BRADOA* *9* *_v* *1* *_* *41390*	Isochorismatase	TGGCACGGCGCTTGC	−37	BRADOA9_v1_41390	BDOA9_RS14875
*phoP*	Alkaline phosphatase synthesis transcriptional regulatory protein	AGGCGGCCGCCCTGC	−233	BRADOA9_v1_21102	BDOA9_RS04660
*BRADOA9_v1_50863*	EF hand or Ca^2+^-binding proteins	TGGCACGGCGCTTGC	−57	BRADOA9_v1_50863	BDOA9_RS28015
*BRADOA9_v1_51520*	Tetratricopeptide repeat protein	AGGAACTCGAATTGT	−50	BRADOA9_v1_51520	BDOA9_RS30760
*BRADOA9_v1_20515*	Signal peptide protein	TGGCGCCTTCCGTGC	−14	BRADOA9_v1_20515	BDOA9_RS35380
*araC*	AraC family transcriptional regulator	TGGACCATCCCATGC	−379	BRADOA9_v1_43245	BDOA9_RS22610
*prkC*	Protein kinase Serine/threonine-protein kinase	CGGCAGGCCGCCCGC	−74	BRADOA9_v1_41482	BDOA9_RS15290
*plk2*	Serine/threonine-protein kinase	TGGATCATTTCTTGC	−69	BRADOA9_v1_p0413	BDOA9_RS34785
*BRADOA9_v1_p0607*	Hemolysin	TGGCACGCCGGTTGC	−47	BRADOA9_v1_p0607	BDOA9_RS35345
*BRADOA9_v1_30300*	Heme-binding protein A	TGGCATCAAGATTGC	−37	BRADOA9_v1_30300	BDOA9_RS09065
*coxS*	Carbon monoxide deHydrogenase small chain	CGGTATACCGCCTGC	−188	BRADOA9_v1_42221	BDOA9_RS18400
*xdhC/coxF*	Xanthine and CO deHydrogenases maturation factor, XdhC/CoxF family	TGGCCCGGCTCTTGC	−60	BRADOA9_v1_51572	BDOA9_RS30985
*dmoA*	Dimethyl-sulfide monooxygenase	TGGACCCATCCGCGC	−149	BRADOA9_v1_40123	BDOA9_RS35475
*BRADOA9_v1_21692*	Putative Propionate CoA-transferase	TGGCGCGGACCTTGC	−110	BRADOA9_v1_21692	BDOA9_RS07125
*BRADOA9_v1_42392*	Glucuronate isomerase	AGGCCAAGTCCCTGC	−51	BRADOA9_v1_42392	BDOA9_RS19090
*phaZ*	PHB_depo_C domain-containing protein	TGGCGTCCTTATTGC	−33	BRADOA9_v1_41018	BDOA9_RS13345
*BRADOA9_v1_51377*	BMP family ABC transporter substrate-binding protein	TGGCACGAAGCTTGC	−73	BRADOA9_v1_51377	BDOA9_RS30150
*yddS*	Putative ABC transporter periplasmic binding protein yddS	TGGGGGAATCGCTGT	−121	BRADOA9_v1_42069	BDOA9_RS17735
*dppE*	Peptide/nickel transport system substrate-binding protein	TGGCATACCATTTGC	−73	BRADOA9_v1_42073	BDOA9_RS17755
*BRADOA9_v1_51377*	Simple sugar transport system substrate-binding protein	TGGCACGAAGCTTGC	−76	BRADOA9_v1_51377	BDOA9_RS30150
*yadG*	Putative ABC transporter ATP-binding protein	TGGCGCCGGCGGTGT	−366	BRADOA9_v1_51886	BDOA9_RS28265
*BRADOA9_v1_40023*	Peptide/nickel transport system substrate-binding protein	TGGCACGAACCTTGC	−43	BRADOA9_v1_40023	BDOA9_RS25085
*dppF*	Dipeptide ABC transporter ATP binding subunit DppF	CGGCCTGCGTAGCGC	−200	BRADOA9_v1_40033	BDOA9_RS09225
*yddS*	Putative ABC transporter periplasmic binding protein	AGGCATACACCTTGC	−49	BRADOA9_v1_40332	BDOA9_RS10465
*BRADOA9_v1_40396*	Extracellular solute-binding protein, family 5	CGGTCGAAACCTCGC	−51	BRADOA9_v1_40396	BDOA9_RS27440
*ydcT*	Putative ABC transporter ATP-binding protein YdcT	TGGCACGGACCTTGC	−40	BRADOA9_v1_50728	BDOA9_RS27435
*gsiA*	Glutathione ABC transporter membrane subunit GsiA	TGGCACGGGAATTGC	−83	BRADOA9_v1_50768	BDOA9_RS09170
*BRADOA9_v1_41971*	Putative aliphatic sulfonates binding protein	CGGCACCGTGATCGC	−355	BRADOA9_v1_41971	BDOA9_RS17315
*braC*	Leucine-, isoleucine-, valine-, threonine-, and alanine-binding protein	CGGAAGAGGTGGTGC	−83	BRADOA9_v1_42172	BDOA9_RS18170
*oppF*	Murein tripeptide ABC transporter/oligopeptide ABC transporter ATP binding subunit OppF	CGGTTGCCGCTTCGC	−132	BRADOA9_v1_41552	BDOA9_RS18395
*BRADOA9_v1_51781*	Hydroxymethylpyrimidine ABC transporter, substrate-binding component	TGGGCATGCTATTGC	−90	BRADOA9_v1_51781	BDOA9_RS19515
*BRADOA9_v1_30301*	ABC transporter substrate-binding protein	TGGCATAGCCATTGC	−51	BRADOA9_v1_30301	BDOA9_RS09070

Note: Both NCBI and Genoscope provided the sequence IDs in the transcriptomic data, whereas only Genoscope provided the sequence IDs for gene analysis, annotation, primer design, and vector construction.

## Data Availability

The original contributions presented in this study are included in the article/Supplementary Material. Further inquiries can be directed to the corresponding author.

## Supplementary Materials

The following supporting information can be downloaded at https://www.mdpi.com/article/10.3390/ijms27104304/s1.
