# Supplementary material for: Unveiling the Functions of Two RpoNs in Bradyrhizobium sp. DOA9 During Free-Living Conditions: A Comprehensive and Comparative Analysis
Source: Int J Mol Sci. 2026 May 12;27(10):4304. doi: 10.3390/ijms27104304 (PMC13207237; doi:10.3390/ijms27104304)
Supplement: Supplementary file 1 [file ijms-27-04304-s001.zip › Supplementary figures and tables legend.pdf]

Supplementary figures/tables legend

Figure S1. Principal component analysis (PCA) chart of metabolomic profiles of *Bradyrhizobium* sp. DOA9 wild type (DOA9WT) and its *rpoN* mutant samples when grown under microaerobically in nitrogen fixing conditions. This shows the clustering relationships between samples: DOA9WT, Δ*rpoNc* (*rpoNc*), and Δ*rpoNp::*Ω*rpoNc* All factors are ranked based on significance; minor factors and noise are eliminated and thereby simplifies the data.

Figure S2. Scatter plot of differential gene KEGG enrichment. X axis: rich factor. Y axis specify KEGG pathways. The size of the dot is positively correlated with the number of differential genes in the pathway. Color code is to indicate different Q value ranges. The smaller the Q value, the more significant the enrichment.

Figure S3. Reverse transcriptase-quantitative PCR (RT-qPCR) analysis of selected genes in four strains under free-living conditions: DOA9WT, Δ*rpoNp*, Δ*rpoNc*, and Δ*rpoNp*::Ω*rpoNc*. Gene expression profiles were assessed using biological triplicates for each condition, focusing on genes encoding proteins involved in predicted metabolic processes and biological pathways in DOA9. The heatmap presents the expression patterns of 40 notable genes, distinguishing RpoNc-regulated genes (A and B) from the genes potentially controlled by both RpoNc and RpoNp (RpoN-regulated genes) (C).

Figure S4. Total down- and up-regulated genes identified from the transcriptome analysis in all comparative datasets.

Figure S5. Organization of gene clusters (operons) potentially regulated by RpoNc, with markers indicating the specific binding positions of RpoN-binding sites upstream of genes involved in each metabolic pathway.

Figure S6. Protein expression, protein purification and investigation of Protein-DNA interaction

Figure S7. Selected genes containing the RpoN-binding site were used to investigate RpoN interaction using Electrophoretic Mobility Shift Assay (EMSA).

Table S1. Venn analysis and result of DEGs based on their comparison group.

Table S2. The specific RpoN binding sites located upstream of genes potentially regulated by either RpoNc or RpoNp are presented, together with gene information categorized by metabolic pathways.

Table S3 Primer used in this study
