# Supplementary material for: Unveiling the Functions of Two RpoNs in Bradyrhizobium sp. DOA9 During Free-Living Conditions: A Comprehensive and Comparative Analysis
Source: Int J Mol Sci. 2026 May 12;27(10):4304. doi: 10.3390/ijms27104304 (PMC13207237; doi:10.3390/ijms27104304)
Supplement: Supplementary file 1 [file ijms-27-04304-s001.zip › Supplementary Methods.pdf]

Supplementary Methods S1

RNA Sequencing

RNA sequencing was performed by GENEWIZ (Azenta Life Sciences) using a standard prokaryotic RNA-seq workflow. Ribosomal RNA (rRNA) was depleted from total RNA using an rRNA removal kit to enrich messenger RNA. Sequencing libraries were constructed using an Illumina-compatible stranded RNA-seq library preparation kit according to the manufacturer's instructions. Libraries were sequenced on an Illumina HiSeq X platform to generate 150-bp paired-end reads. Across biological triplicates, approximately 21–26 million paired-end reads per sample were obtained. Base calling and demultiplexing were performed using Bcl2FastQ (v2.17.1.14). Raw data quality was assessed using FastQC.

Bioinformatics Analysis

Raw reads were processed using a standard RNA-seq analysis pipeline. Adapter sequences and low-quality bases were trimmed using Cutadapt (v1.9.1). Clean reads were then aligned to the *Bradyrhizobium* sp. DOA9 reference genome (ASM61784v2) using Bowtie2 (v2.2.6) with default parameters.

Aligned reads were assigned to annotated genes using HTSeq-count (v0.6.1) to generate gene-level count matrices. Differential gene expression analysis was performed in DESeq2 (v1.38.0) within the R environment. Genes were considered significantly differentially expressed when they met the following criteria: (1) adjusted p-value (Benjamini–Hochberg FDR) < 0.05, and (2) absolute $\log_2$ fold change ≥ 1 (equivalent to ≥2-fold change). Volcano plots and heatmaps were generated using R.

Functional Enrichment Analysis

Functional enrichment analysis of differentially expressed genes was performed using GOseq to identify significantly enriched Gene Ontology (GO) terms while correcting for gene length bias. KEGG pathway enrichment analysis was performed to identify significantly affected metabolic and cellular pathways. Enrichment results with FDR ≤ 0.05 were considered statistically significant.

Supplementary Methods S2

Stress tolerances drop-plate assay and survival analysis

To investigate the role of the *rpoN* genes in bacterial adaptation and survival under environmental stress conditions, a drop-plate assay was performed using *Bradyrhizobium* sp. DOA9 wild-type (WT) and the mutant strains (∆*rpoNc*, ∆*rpoNp*, and ∆*rpoNp*::Ω*rpoNc*). Cultures were grown in YEM medium at 30 °C for 5 days until reaching an optical density of OD_{600} ≈ 1.0 (approximately 1 × 10^8 cells mL^{-1}). Ten-fold serial dilutions were prepared, and 10 µL of each dilution was spotted onto YEM agar plates under the following conditions: acidic (pH 5.0), alkaline (pH 8.8), and elevated temperature (37 °C). The pH of the media was adjusted using 2 N HCl or 5 N NaOH, while untreated YEM plates incubated at 30 °C served as the control condition. Plates were incubated for 7 days prior to assessment of bacterial growth.

For quantitative evaluation, percent survival was calculated based on viable colony counts (CFU). Colonies from appropriate dilution plates were counted, and survival under each stress condition was expressed relative to the control condition according to the formula: survival (%) = (CFU under stress / CFU under control) × 100. All experiments were performed using biological replicates to ensure reproducibility.
