# Supplementary material for: Unveiling the Functions of Two RpoNs in Bradyrhizobium sp. DOA9 During Free-Living Conditions: A Comprehensive and Comparative Analysis
Source: Int J Mol Sci. 2026 May 12;27(10):4304. doi: 10.3390/ijms27104304 (PMC13207237; doi:10.3390/ijms27104304)
Supplement: Supplementary file 1 [file ijms-27-04304-s001.zip › Table S2.pdf]

Table S3. The specific RpoN binding sites located upstream of genes potentially regulated by either RpoNc or RpoNp are presented, together with gene information categorized by metabolic pathways.

**Metabolite/Gene**	**Description**	**Prediction of RpoN-binding site**	**Position**	**Sequence ID (Genoscope)**	**Sequence ID (NCBI)**
groS	Cochaperonin GroES	TGGATGTTGACGTGC	-331	BRADOA9_v1_41333	BDOA9_RS14650
padR	Environmental sensors. PadR	CGGCTTGCGGCGTGT	-301	BRADOA9_v1_30093	BDOA9_RS08240
BRADOA9_v1_40926	DUF465 domain-containing protein	CGGCATGTGCCTTGC	-32	BRADOA9_v1_40926	BDOA9_0125680
hrp	Hypoxic response protein 1	CGGGCGCTTCAGCGC	-46	BRADOA9_v1_20309	BDOA9_RS01260
BRADOA9_v1_20612	DUF4169 family	CGGGTCGTATTGCGT	-65	BRADOA9_v1_20612	BDOA9_RS02540
mscS	MscS Mechanosensitive ion channel	CGGGAGGCGTTGTGC	-33	BRADOA9_v1_42478	BDOA9_RS19445
selO	Protein adenylyltransferase SelO	TGGCACTCCCCTTGC	-59	BRADOA9_v1_20586	BDOA9_RS02420
BRADOA9_v1_42396	TerB family tellurite resistance protein	CGGGCCCCTGTTCGT	-56	BRADOA9_v1_42396	BDOA9_RS19110
norM	Probable multidrug resistance protein NorM	CGGCAGGCGAGGCGC	-116	BRADOA9_v1_21673	BDOA9_RS07035
bfrD	*tonB*-dependent receptor BfrD	AGGAAGATGGGGCGT	-7	BRADOA9_v1_42528	BDOA9_RS19665
BRADOA9_v1_20612	DUF4169 family protein	CGGGTCGTATTGCGT	-62	BRADOA9_v1_20612	BDOA9_RS02540
BRADOA9_v1_50952	Trigger factor	TGGATGATGTTGCGT	-206	BRADOA9_v1_50952	BDOA9_RS28395
BRADOA9_v1_21519	DNA topoisomerase IB	CGGAAGGCTCGTTGT	-241	BRADOA9_v1_21519	BDOA9_RS06375
BRADOA9_v1_40052	3-oxoacyl reductase	TGGCTGGTCGTTCGC	-47	BRADOA9_v1_40052	BDOA9_RS09310
serA	Phosphoglycerate dehydrogenase	CGGATGACGTGCCGT	-32	BRADOA9_v1_20385	BDOA9_RS01560
pckA	Phosphoenolpyruvate carboxykinase (ATP)	TGGCAACGCCAGCGT	-112	BRADOA9_v1_20821	BDOA9_RS03455
BRADOA9_v1_20901	MFS domain-containing protein	CGGGCATGACGGCGC	-19	BRADOA9_v1_20901	BDOA9_RS03820
BRADOA9_v1_51082	EGF-like domain; Glycoprotein	TGGCGCCGGATTTGC	-119	BRADOA9_v1_51082	BDOA9_RS28910
BRADOA9_v1_20355	Cupin type-1 domain-containing protein	CGGCAGCGCCCGCGT	-265	BRADOA9_v1_20355	BDOA9_RS01445
BRADOA9_v1_41342	Putative Sulfur globule protein CV2	AGGCCCGCCGCTTGC	-27	BRADOA9_v1_41342	BDOA9_RS14690
htpG	Chaperone protein HtpG	AGGTCGCAAGGCTGC	-139	BRADOA9_v1_20505	BDOA9_RS02055
BRADOA9_v1_20115	Adenylate cyclase domain	TGGCGCCGTCGATGC	-106	BRADOA9_v1_20115	BDOA9_RS00455

Note: Both NCBI and Genoscope provided the sequence IDs in the transcriptomic data, whereas only Genoscope provided the sequence IDs for gene analysis, annotation, primer design, and vector construction.
