# Supplementary material for: Unveiling the Functions of Two RpoNs in Bradyrhizobium sp. DOA9 During Free-Living Conditions: A Comprehensive and Comparative Analysis
Source: Int J Mol Sci. 2026 May 12;27(10):4304. doi: 10.3390/ijms27104304 (PMC13207237; doi:10.3390/ijms27104304)
Supplement: Supplementary file 1 [file ijms-27-04304-s001.zip › Table S3.pdf]

Table S2. Primer used in this study

Target gene	Sequence ID (Genoscope)	Sequence ID (NCBI)	Primer name	Sequence (5' to 3')	Description
BRADOA9_v1_50925	BRADOA9_v1_50925	BDOA9_RS28285	thio.qRT.F<br>thio.qRT.R	GACGAGTGGATCGGCGATAG<br>TAGAGATAGACCGGCGTGGT	Iron-sulfate metabolism
erpA	BRADOA9_v1_43530	BDOA9_RS30765	erpA.qRT.F<br>erpA.qRT.R	GACTGCCGTGACCATCAG<br>CGGAATCGACCAGCACC	
hyaAc	BRADOA9_v1_42129	BDOA9_RS18000	hyaAc.qRT.F<br>hyaAc.qRT.R	CGTCCGAAGATGTTCTACTCG<br>ACTGCTTGATGTTGGTGAGG	Hydrogenase
hyaAp	BRADOA9_v1_p0690	BDOA9_RS35605	hyaAp.qRT.F<br>hyaAp.qRT.R	CGCGTCGAAGCTTTCACAAA<br>GAGCGAGATCATCGACAGCA	
BRADOA9_v1_41488	BRADOA9_v1_41488	BDOA9_RS15315	heamo.qRT.F<br>heamo.qRT.R	GAGCAGATTACCCTCGTTCAG<br>ATAGTCGACGTGACGCTTG	Hemoglobin
hemA	BRADOA9_v1_30300	BDOA9_RS09065	hemA.qRT.F<br>hemA.qRT.R	ATGCGGTGATCTGGAATCTG<br>CAATCCTTGCCGAGCTTTTC	
BRADOA9_v1_51526	BRADOA9_v1_51526	BDOA9_RS30785	51526.qRT.F<br>51526.qRT.R	GGCGAGGGAAGAGATCGAC<br>ATCCTGCGTGAAGAAGTCGC	Electron exchange
fdx	BRADOA9_v1_51516	BDOA9_RS30745	fdx.qRT.F<br>fdx.qRT.R	ACGTCTTCGCCTGCCATAC<br>ACCAGACACCGTCGGGATA	
glnII	BRADOA9_v1_42831	BDOA9_RS20900	glnII.qRT.F<br>glnII.qRT.R	CAACTTCTCGACCGCCTATATG<br>CTTCCAGATAGCCCTTGTAGC	GS and GOGAT
glnB	BRADOA9_v1_50070	BDOA9_RS24700	glnB.qRT.F<br>glnB.qRT.R	TCAGGAAGTCGGTCTCCAG<br>CGCCTCTTCGATGTTGGAG	
gltK	BRADOA9_v1_40129	BDOA9_RS09625	gltK.qRT.F<br>gltK.qRT.R	CACTGCCCTATCTGTTCTACG<br>CACCAGGAAGTAGAACCAGAAG	Glutamate
urtA	BRADOA9_v1_40426	BDOA9_RS10850	urtA.qRT.F<br>urtA.qRT.R	GCCTACACCAAGAATCCGAAG<br>TCTTCCAAACCACGTCGAAC	Urea
nrtA	BRADOA9_v1_41484	BDOA9_RS15300	nrtA.qRT.F<br>nrtA.qRT.R	AAGGACTGGAAGGGAATGAAG<br>TGGAGAGCAGATGCATGAAG	Nitrate
fnt	BRADOA9_v1_41483	BDOA9_RS15295	fnt.qRT.F<br>fnt.qRT.R	TCGGAATTCGTCACCAAGATG<br>CCAGGAGATAGAGCATGACG	
phaZ	BRADOA9_v1_41018	BDOA9_RS13345	phaZ.qRT.F<br>phaZ.qRT.R	ATCATGTGCGAGGACAACC<br>CGAAGCCGGGATAGACTTTC	Cabon
mglA	BRADOA9_v1_51381	BDOA9_RS30170	mglA.qRT.F<br>mglA.qRT.R	TCGTCAAATGCATCATGGGC<br>CATCTCACTCGGCCAGTTCA	
atzE	BRADOA9_v1_40018	BDOA9_RS09160	atzE.qRT.F<br>atzE.qRT.R	GCACCCTCAACTCCTTCAC<br>CTCCATCCGCTCGATCAG	Amino acid metabolism
thiO	BRADOA9_v1_41077	BDOA9_RS13585	thiO.qRT.F<br>thiO.qRT.R	CATGACCGAGCATCACCG<br>GATCACGATGCCGTCTAGTTC	
YdcT	BRADOA9_v1_50728	BDOA9_RS27435	YdcT.qRT.F<br>YdcT.qRT.R	AATATGCAGGCCGTCTTCC<br>TGACCTGGATGAAGCTGATG	Spermidine
potC	BRADOA9_v1_42218	BDOA9_RS27450	potC.qRT.F<br>potC.qRT.R	GGACGTTCTATGTCCTGGCA<br>GTCGAACGAGCCCTTGACAT	
CoA-transferase	BRADOA9_v1_21692	BDOA9_RS07125	CoA-.qRT.F<br>CoA-.qRT.R	CTGCTCTACAAGACCTTTCCG<br>GGAATCTTGACCTGGCGG	TCA
Glucuronate isomerase	BRADOA9_v1_42392	BDOA9_RS19090	42392.qRT.F<br>42392.qRT.R	GTCCCACCGATTTCCATCTG<br>AGGAATTTGCCGGAATAGCC	
cheY	BRADOA9_v1_51515	BDOA9_RS30735	cheY.qRT.F<br>cheY.qRT.R	ACGACGAAATCGAGACGCAC<br>CTCGACCGCAATTGTCTCGT	Motility
flgF	BRADOA9_v1_51018	BDOA9_RS28660	flgF.qRT.F<br>flgF.qRT.R	GACCATTCGCTGTTCGAGGAT<br>CGTCGCGGGTATAGAACTCG	
PliZ	BRADOA9_v1_43052	BDOA9_RS21810	PliZ.qRT.F<br>PliZ.qRT.R	AGTGGATGTTGCGGTCAG<br>CTGATGGTGCATTGGAATTCG	Cellular surface polysaccharide
spsC	BRADOA9_v1_51531	BDOA9_RS30800	spsC.qRT.F<br>spsC.qRT.R	CACGACTTCATTCCGCTCTC<br>AACGAATAGGACGAAGCGAC	

shc	BRADOA9_v1_41724	BDOA9_RS16295	shc.qRT.F<br>shc.qRT.R	AGCGTGAAGGCGTATTTCG<br>TGTTGAGATGGAACGGCG	
bolA	BRADOA9_v1_20940	BDOA9_RS30755	bolA.qRT.F<br>bolA.qRT.R	GGACCGGAGATCGAGCAGT<br>GCCTCGCCCATTTGTCCTTTC	Growth and cell division
rpsE	BRADOA9_v1_50507	BDOA9_RS26510	rpsE.qRT.F<br>rpsE.qRT.R	AAGCTCGTCCACATCAACC<br>GCACGCAGATAGACTCGG	
rluD	BRADOA9_v1_20316	BDOA9_RS34655	rluD.qRT.F<br>rluD.qRT.R	GCATTGGGAAATCCTGGAAAG<br>AGCAGGTAAGCATGTAAGGC	
BRADOA9_v1_p0683	BRADOA9_v1_p0683	BDOA9_RS35575	p0683.qRT.F<br>p0683.qRT.R	TCGTATCCTCGTCTATCCCG<br>GGAAATACTGGTTGGCTTTGC	Responsive system
mtrA	BRADOA9_v1_50919	BDOA9_RS28260	mtrA.qRT.F<br>mtrA.qRT.R	GGAAACACATCTGCGAGAAG<br>GTTCACCATCACCATCTCGTAG	
cynS	BRADOA9_v1_21776	BDOA9_RS07490	cynS.qRT.F<br>cynS.qRT.R	CATCGACCAGATCACGCC<br>CATTGCCGAGATGAACGC	
BRADOA9_v1_41390	BRADOA9_v1_41390	BDOA9_RS14875	Iso.qRT.F<br>Iso.qRT.R	CTTCCACATCATCCATACCCG<br>GATCAGCTCCAGATCGGTG	Infection and Virulence
phoP	BRADOA9_v1_21102	BDOA9_RS04660	phoP.qRT.F<br>phoP.qRT.R	GTCTACCTCCAACGCAACA<br>CGAGTTCAGGTTTGCGAAAG	
BRADOA9_v1_50863	BRADOA9_v1_50863	BDOA9_RS28015	EF hand.qRT.F<br>EF hand.qRT.R	GACCAATCCGTTCGCAATC<br>ACTCGGACTTGGTAATCTTGC	
plK2	BRADOA9_v1_p0413	BDOA9_RS34785	plK2.qRT.F<br>plK2.qRT.R	GCAAACGCCGGTATACATTTC<br>CATCTAATCGGCCTACAGTCAC	
traI	BRADOA9_v1_p0112	BDOA9_RS33765	traI.qRT.F<br>traI.qRT.R	AAGCCGACTTATCTCTTGGTG<br>ATTCAATCATGGCCGCAAAG	Secretion system
coxF	BRADOA9_v1_51572	BDOA9_RS30985	coxF.qRT.F<br>coxF.qRT.R	GCCACATCGACCTTCTGATC<br>ACATCACCCAGCGAACTTG	Regulates atmospheric CO and CO2 levels
coxS	BRADOA9_v1_42221	BDOA9_RS18400	coxS.qRT.F<br>coxS.qRT.R	GGACGTTCTATGTCCTGGCA<br>GTCGAACGAGCCCTTGACAT	
gst	BRADOA9_v1_41019	BDOA9_RS13350	gst.qRT.F<br>gst.qRT.R	TTGCCGAATATCTCAACGAGG<br>CCGATTTCTCCAGGCAGTC	
BRADOA9_v1_50859	BRADOA9_v1_50859	BDOA9_RS28000	50859.qRT.F<br>50859.qRT.R	TCCGGGTCAATCGTTTCGAG<br>GGGGCGATTGGTCGATGAAA	Motility
cheB	BRADOA9_v1_50313	BDOA9_RS25710	cheB.qRT.F<br>cheB.qRT.R	ATCATTGTCATCGGGGGCTC<br>CCCAGATAGACATGACCCGC	
BRADOA9_v1_20912	BRADOA9_v1_20912	BDOA9_RS03870	20912.qRT.F<br>20912.qRT.R	GCACCTACAGCTACGTCCTC<br>ATCACGGTCATGCAATGGGT	Fe-S
ybiX	BRADOA9_v1_42529	BDOA9_RS19670	ybiX.qRT.F<br>ybiX.qRT.R	GAAAACTCGGCAATCGCGTC<br>GATCGACAAGTCTGTGCGGA	
CV2	BRADOA9_v1_41342	BDOA9_RS14690	CV2.qRT.F<br>CV2.qRT.R	CTTGCTCTTGCAATCGCCG<br>GACCATAGACCGGCGCATAA	
fpr	BRADOA9_v1_42471		fpr.qRT.F<br>fpr.qRT.R	GATCGACAATCTGGAAGAGGG<br>CACGGTCGGGTAATAGATCAG	
gatA	BRADOA9_v1_40035	BDOA9_RS09235	gatA.qRT.F<br>gatA.qRT.R	CTTTGATGACGCTCACCGAG<br>ATACATGTCCTTGTGCGCGA	Glutamate
BRADOA9_v1_20355	BRADOA9_v1_20355	BDOA9_RS01445	20355.qRT.F<br>20355.qRT.R	CGCAACGGCGTCTACGATTA<br>CGATCACGCAGAAATCCTCG	CSP
BRADOA9_v1_51767	BRADOA9_v1_51767	BDOA9_RS31825	51767.qRT.F<br>51767.qRT.R	AGCGGAGAGAAGTTCGACAAG<br>CCTCGAGCCTGACATTGGTG	
BRADOA9_v1_20901	BRADOA9_v1_20901	BDOA9_RS03820	20901.qRT.F<br>20901.qRT.R	CTCTATCTCGCCAATGCCGT<br>CATACATCGCACCGGCAAAG	
pckA	BRADOA9_v1_20821	BDOA9_RS03455	pckA.qRT.F<br>pckA.qRT.R	AAATCGGTCTTCACCACGCT<br>GACAGCTTGATGCACTTGGC	TCA
serA	BRADOA9_v1_20385	BDOA9_RS01560	serA.qRT.F<br>serA.qRT.R	CCGTTCGGCAATTCGATCAC<br>GAACGCGATCACCTTCATGC	
BRADOA9_v1_40052	BRADOA9_v1_40052	BDOA9_RS09310	40052.qRT.F	TCAAGGGCTATGCGCAGTC	Fatty acid

			40052.qRT.R	CTGCAGCACGTTCCAATAGC	
echA8	BRADOA9_v1_40051	BDOA9_RS09305	echA8.qRT.F<br>echA8.qRT.R	GCGAATTGATGTTCACCGGC<br>TTCGAAGTCGAACAGCAGGG	
BRADOA9_v1_21519	BRADOA9_v1_21519	BDOA9_RS06375	21519.qRT.F<br>21519.qRT.R	GGCAAGGGCTATTCCTTCGT<br>TCCCAATCGGCATGATAGCG	Growth and Cell division
smc1a	BRADOA9_v1_30257	BDOA9_RS08925	smc1a.qRT.F<br>smc1a.qRT.R	GACGACGTCGCCAAAGCC<br>CTCCTCCAGCTCCGACAAA	
BRADOA9_v1_p0875	BRADOA9_v1_p0875	BDOA9_RS36485	p0875.qRT.F<br>p0875.qRT.R	AAGACCCCTGATGCCGATCT<br>CTTGCGCAGCTTGGGAATAC	
norM	BRADOA9_v1_21673	BDOA9_RS07035	norM.qRT.F<br>norM.qRT.R	CATCATTCCGGTCTTGCCGA<br>ACCCCGACACGAAATACCAG	Responsive system
groL	BRADOA9_v1_41335	BDOA9_RS14655	groL.qRT.F<br>groL.qRT.R	GTCGCCTCGAAGACCAATGA<br>AGGTGATCTTCTTGGCGTGG	
BRADOA9_v1_20118	BRADOA9_v1_20118	BDOA9_RS00465	20118.qRT.F<br>20118.qRT.R	TCATCGACTGGCTGACCAAC<br>GCCGATATATTCGGACCGCT	
selO	BRADOA9_v1_20586	BDOA9_RS02420	selO.qRT.F<br>selO.qRT.R	ATGCAGGAAGTGTCCCGTTC<br>ATCGTCGCGACCAGATTGT	
bepG	BRADOA9_v1_20922	BDOA9_RS03915	bepG.qRT.F<br>bepG.qRT.R	TTGGCCGTCTCTCCATCAAC<br>ATACAGCATGTCCTCGACGC	Stress response and quorum sensing
HspC1	BRADOA9_v1_40922	BDOA9_RS12940	HspC1.qRT.F<br>HspC1.qRT.R	GGTCGAGAAACCAGGAGCTT<br>GAACGTCCTTCTCGGTCAGG	
BRADOA9_v1_20115	BRADOA9_v1_20115	BDOA9_RS00455	20115.qRT.F<br>20115.qRT.R	AACGTCTCCGGATACACGTC<br>GATCGCGGCCTTGGTGTAG	
htpG	BRADOA9_v1_20505	BDOA9_RS02055	htpG.qRT.F<br>htpG.qRT.R	CTGTGCATACGCAGCCTTTC<br>TTGGCCGTCTTGTTCGGAAT	
htrA	BRADOA9_v1_41386	BDOA9_RS14860	htrA.qRT.F<br>htrA.qRT.R	CTGCTGGACGCCTATTCCAA<br>GATCGATCCCGAGCACCTG	
qheDH	BRADOA9_v1_21033	BDOA9_RS04385	qheDH.qRT.F<br>qheDH.qRT.R	TGGAACGAAGGGCTTCTTGG<br>GAAGGAGTAAGCGAGCGTCA	
rpoNc	BRADOA9_v1_21339	BDOA9_RS05640	RpoNc.BamHI.F<br>RpoNc.EcoRI.R	GGATCCATGGCGCTTACGCAGAGATT<br>GAATTCCAGGCCGGTTCGGGATTGCG	Primers were designed to amplify the rpoNc fragment, which was subsequently cloned into the pET22(+) vector and expressed in Escherichia coli BL21(+) cells.
rpoNp	BRADOA9_v1_p0436	BDOA9_RS37280	RpoNp.NcoI.F<br>RpoNp.SalI.R	CCATGGAATGAGCCATGCATATCAAC<br>GTCGACGTCGTCTCTAATCTGTTCTG	Primers were designed to amplify the rpoNp fragment, which was subsequently cloned into the pET22(+) vector and expressed in Escherichia coli BL21(+) cells.
pmrpoNc	BRADOA9_v1_21339	BDOA9_RS05640	pmrpoNc.F<br>pmrpoNc.R	CATCCGTCGACTCGTCCGCCATCTGACCAATC<br>AATCTTCTAGAAAGCGCCATGAAGCAAGAACC	For analysis of the interaction between RpoN and the promoter region (pm) of the target gene.
pmrpoNp	BRADOA9_v1_p0436	BDOA9_RS37280	pmrpoNp.F<br>pmrpoNp.R	CTGCGGTCGACCGTCGCAAAACTACCTGCAC<br>CCTGTTCTAGAGCATGGCTCATTGAAACACCTTC	For analysis of the interaction between RpoN and the promoter region (pm) of the target gene.
pmntrA	BRADOA9_v1_50923	BDOA9_RS28275	pmntrA.300bp.F<br>pmntrA.300bp.F	ATGCAGATCGCAAATTTTGC<br>TAGGCAGTGTCGTCCTTTGG	For analysis of the interaction between RpoN and the promoter region (pm) of the target gene.
pmtraI	BRADOA9_v1_p0112	BDOA9_RS33765	pmtraIp.300bp.F<br>pmtraIp.300bp.R	TCTGTCAGAGTTGCAGCATC<br>TTCATAACCTCAGGCTTACG	For analysis of the interaction between RpoN and the promoter region (pm) of the target gene. .
pmnifB	BRADOA9_v1_51543	BDOA9_RS30845	pmnifB.CSP.300bp.F<br>pmnifB.CSP.300bp.R	GATCGTGCTCGCGGGCTGAC<br>GTTCTCCTTGCAGGCCGAAC	For analysis of the interaction between RpoN and the promoter region (pm) of the target gene.
pmgltJ	BRADOA9_v1_40129	BDOA9_RS09625	pmgltK.300bp.F	CGGCGTTCAAGAAGGTCGTC	For analysis of the interaction between RpoN and the promoter region (pm) of the target gene.

			pmgltK.300bp.R	GAACGCGCCAGCCATAGATG	
nodA1	BRADOA9_v1_51377	BDOA9_RS30150	pmABC trans.F<br>pmABC trans.R	GAGCTTTCCATCTTCTGGGGA<br>AGGCTACAAAGAGAGCAGAACT	For analysis of the interaction between RpoN and the promoter region (pm) of the target gene.
	BRADOA9_v1_p0375	-	nodA1.F<br>nodA1.R	CGACCATGCGGGGAGAACAAAG<br>GTGTGGCAGGCAATGTGGGATAG	It was used as negative control for analysis of the interaction between RpoN and the promoter region (pm) of the target gene.

Noted. More information of these target genes was described in the Table S1.
