# Supplementary figures and images for: Unveiling the Functions of Two RpoNs in Bradyrhizobium sp. DOA9 During Free-Living Conditions: A Comprehensive and Comparative Analysis
Source: Int J Mol Sci. 2026 May 12;27(10):4304. doi: 10.3390/ijms27104304 (PMC13207237; doi:10.3390/ijms27104304)

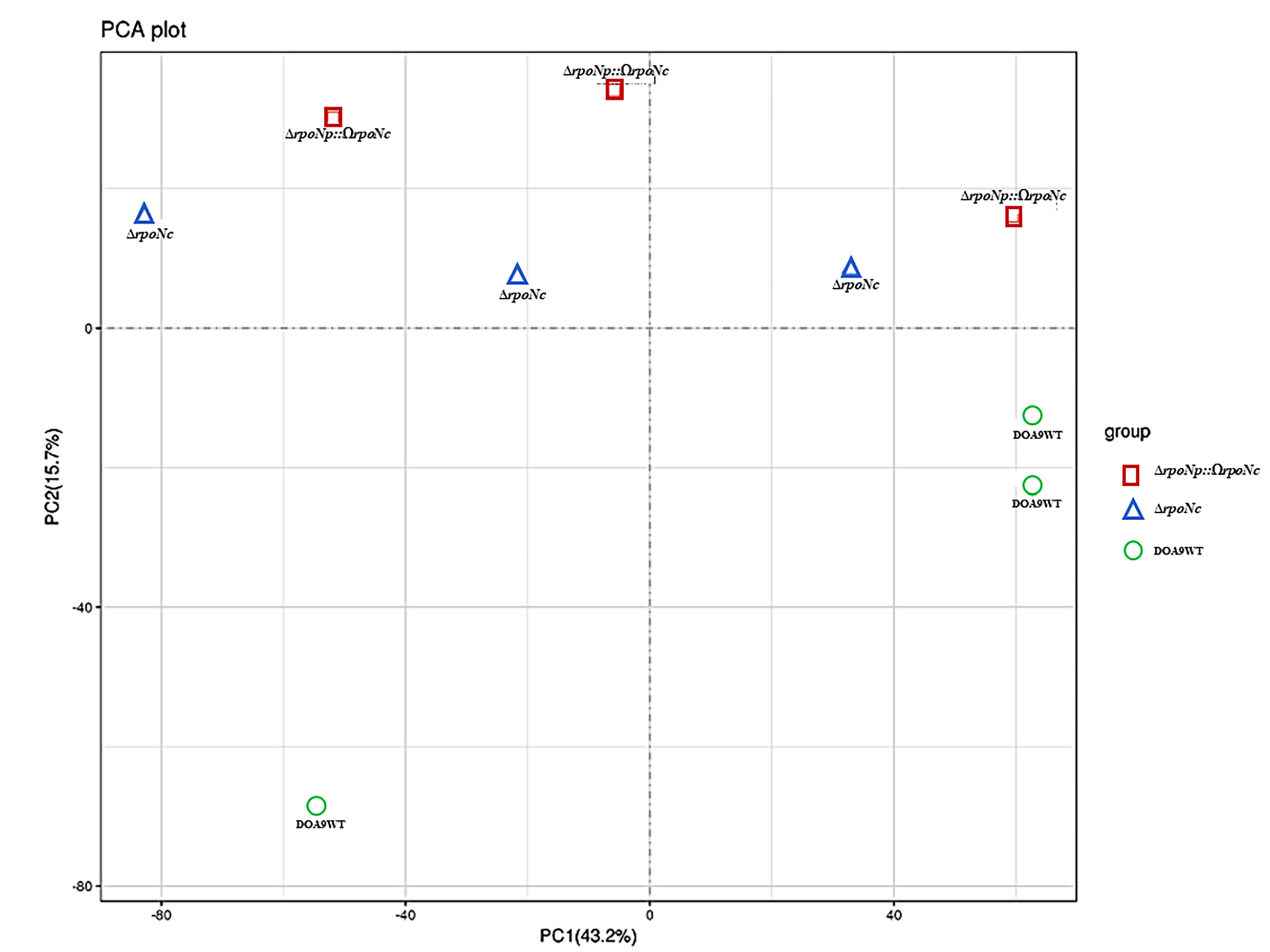

Supplement: Supplementary file 1 [file ijms-27-04304-s001.zip › Figure S1.png]

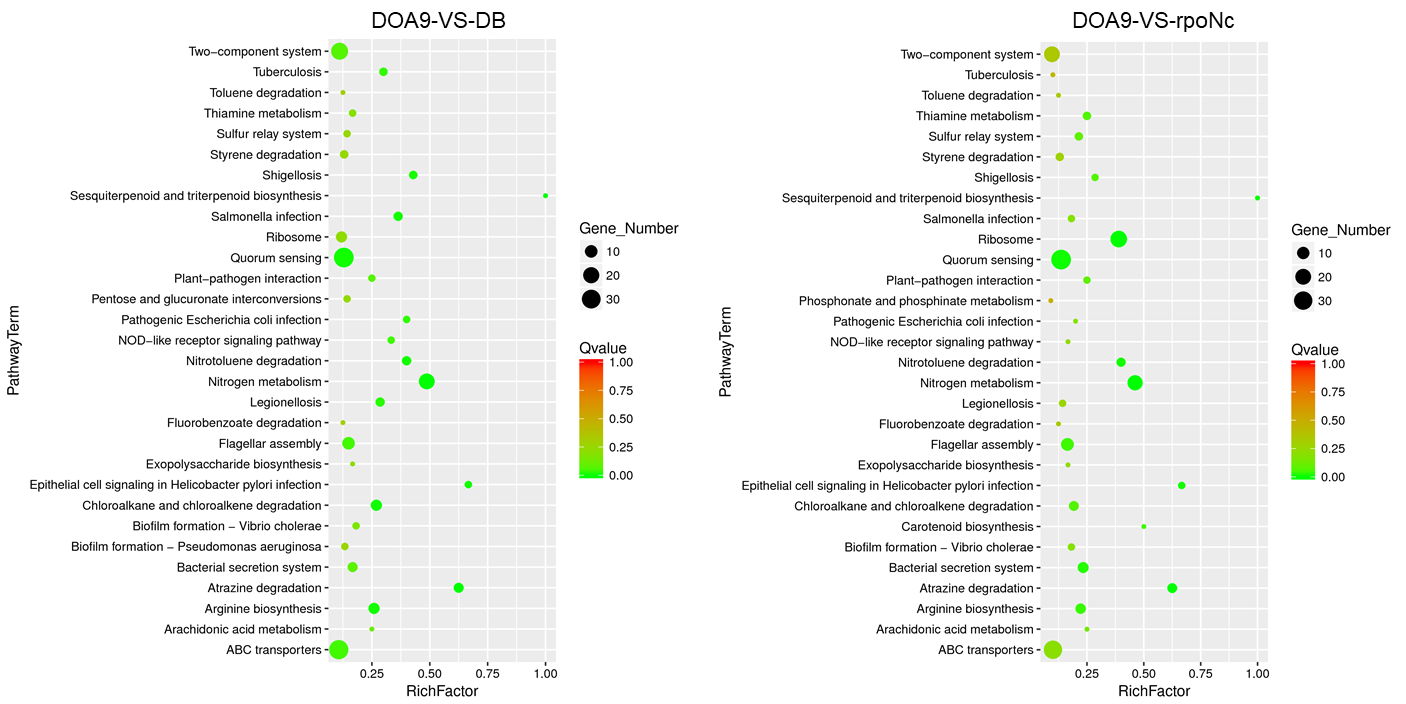

Supplement: Supplementary file 1 [file ijms-27-04304-s001.zip › Figure S2.png]

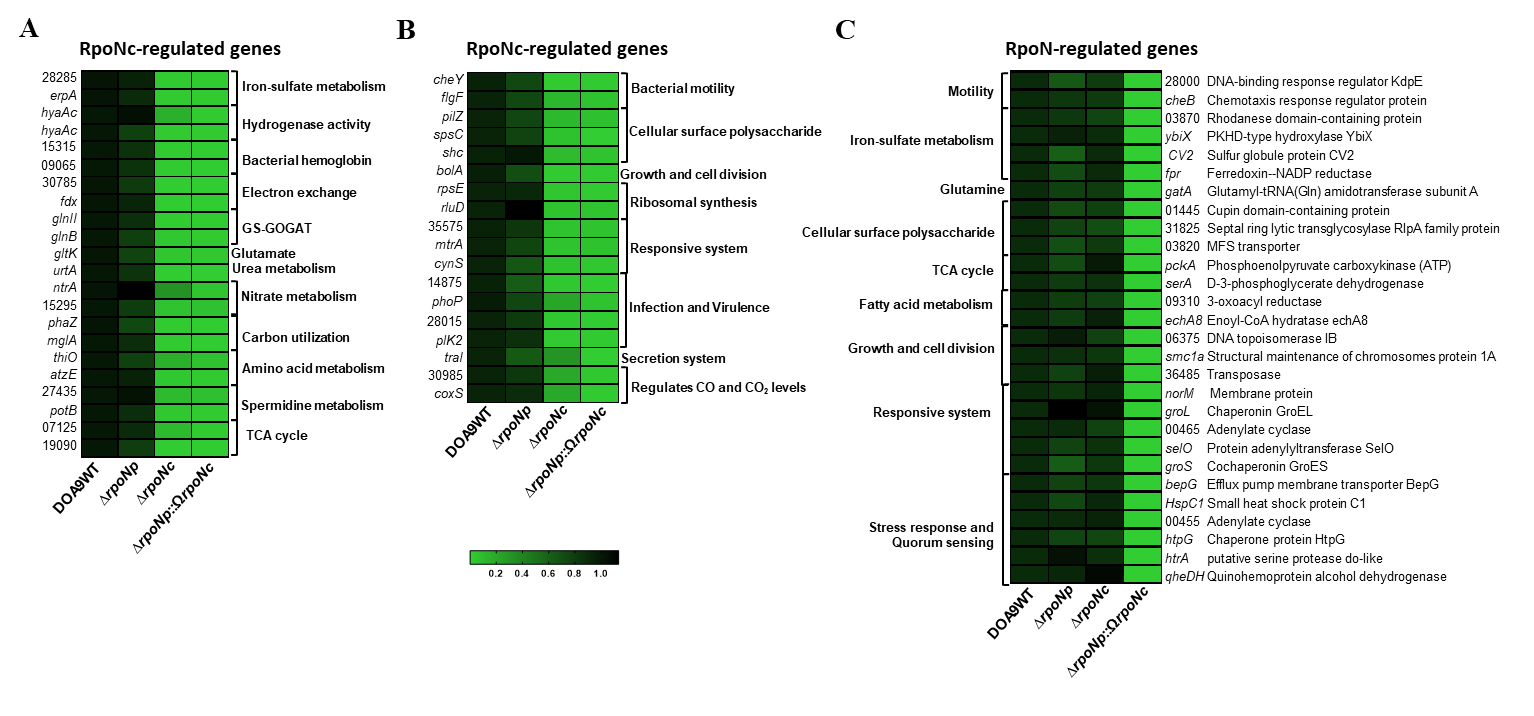

Supplement: Supplementary file 1 [file ijms-27-04304-s001.zip › Figure S3.png]

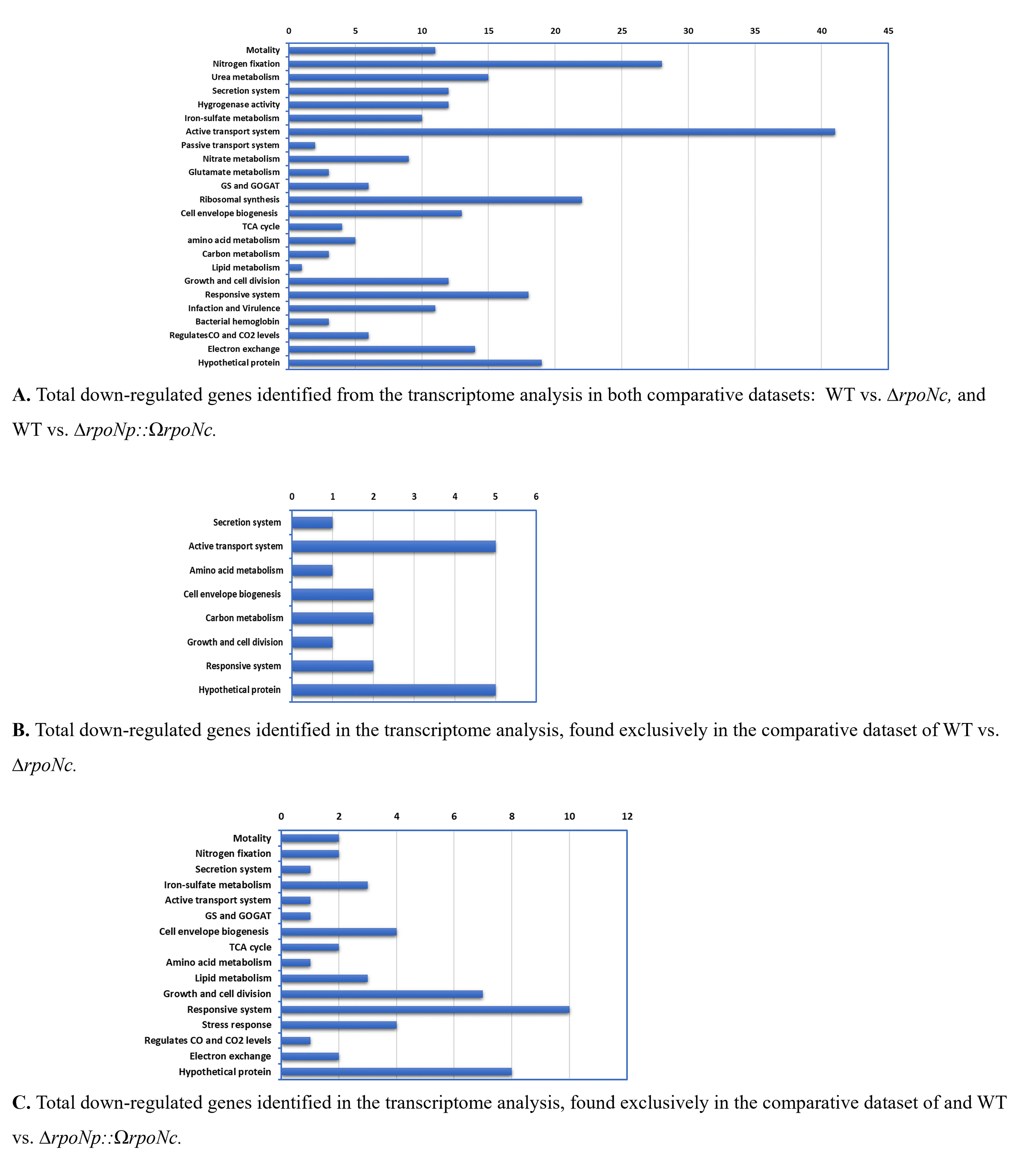

Supplement: Supplementary file 1 [file ijms-27-04304-s001.zip › Figure S4A-C.png]

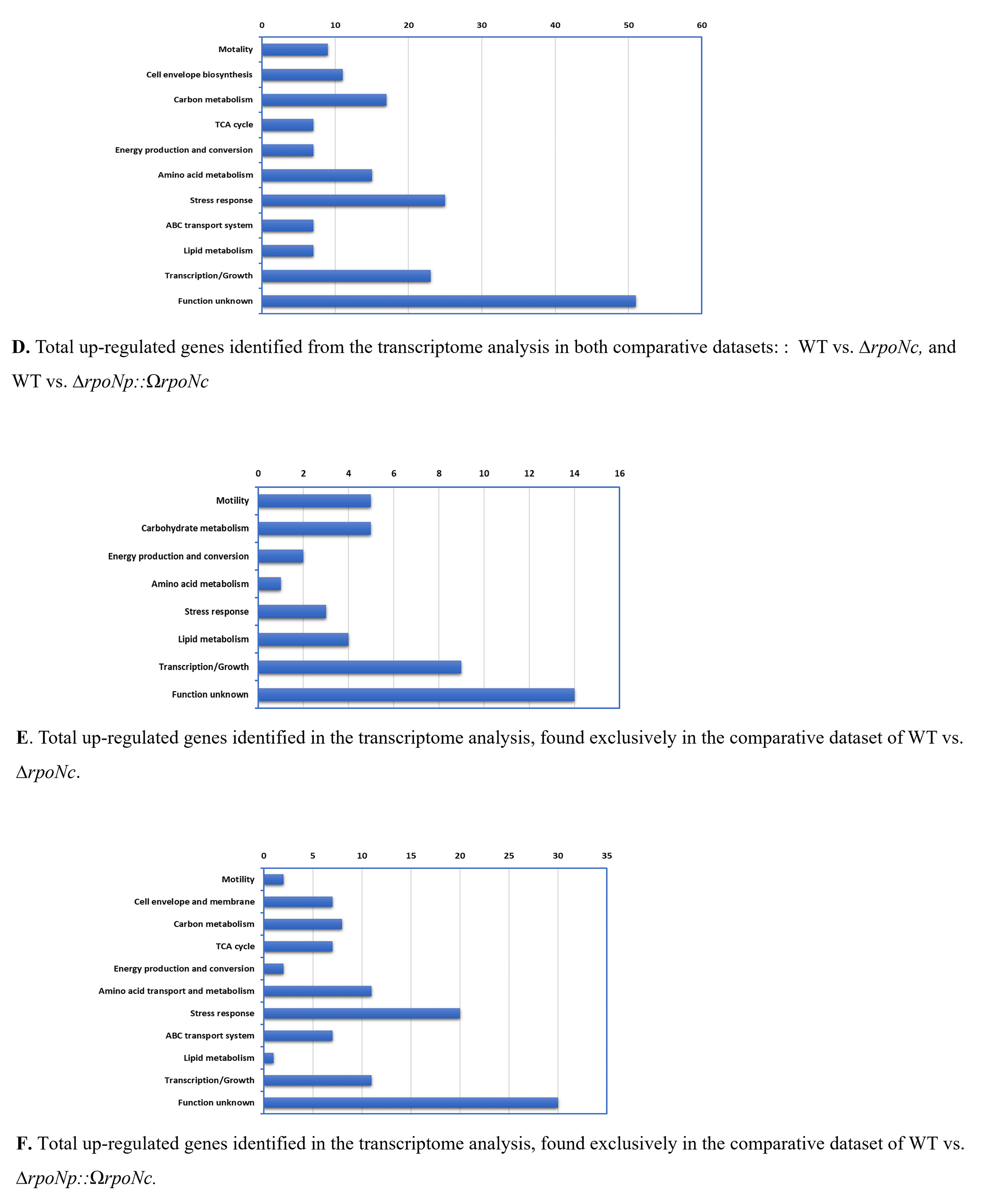

Supplement: Supplementary file 1 [file ijms-27-04304-s001.zip › Figure S4D-F.png]

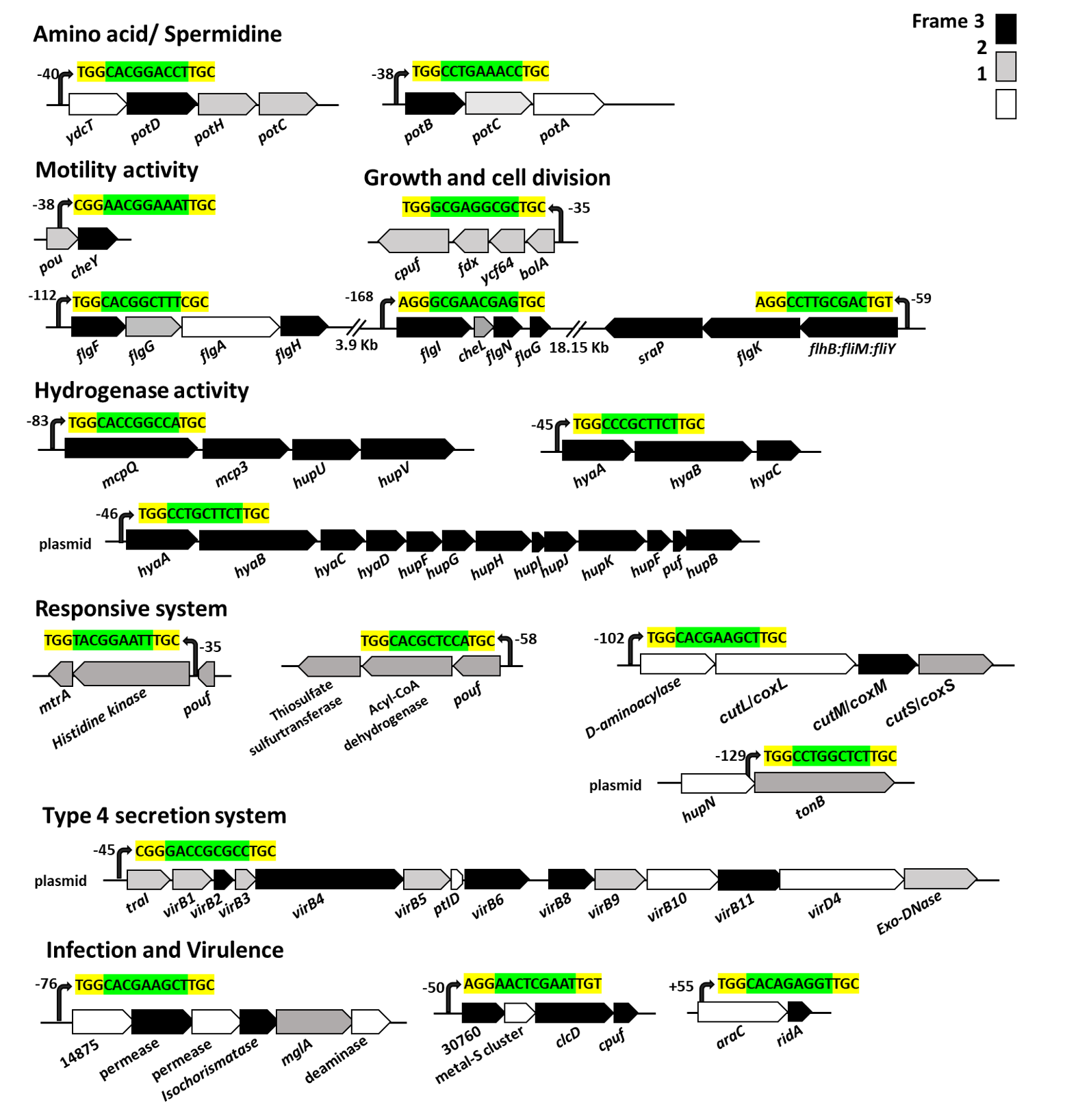

Supplement: Supplementary file 1 [file ijms-27-04304-s001.zip › Figure S5-continued.png]

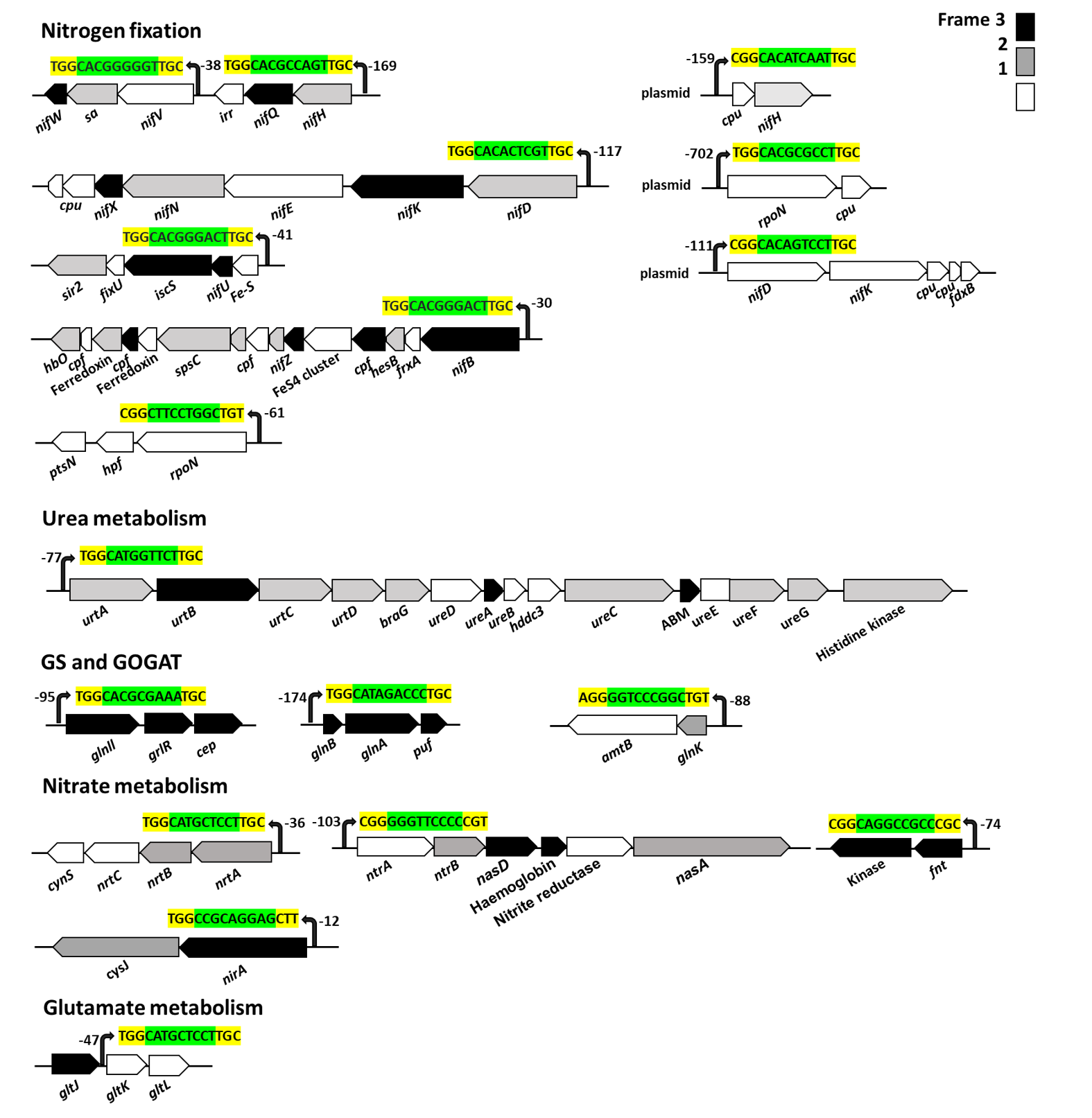

Supplement: Supplementary file 1 [file ijms-27-04304-s001.zip › Figure S5.png]

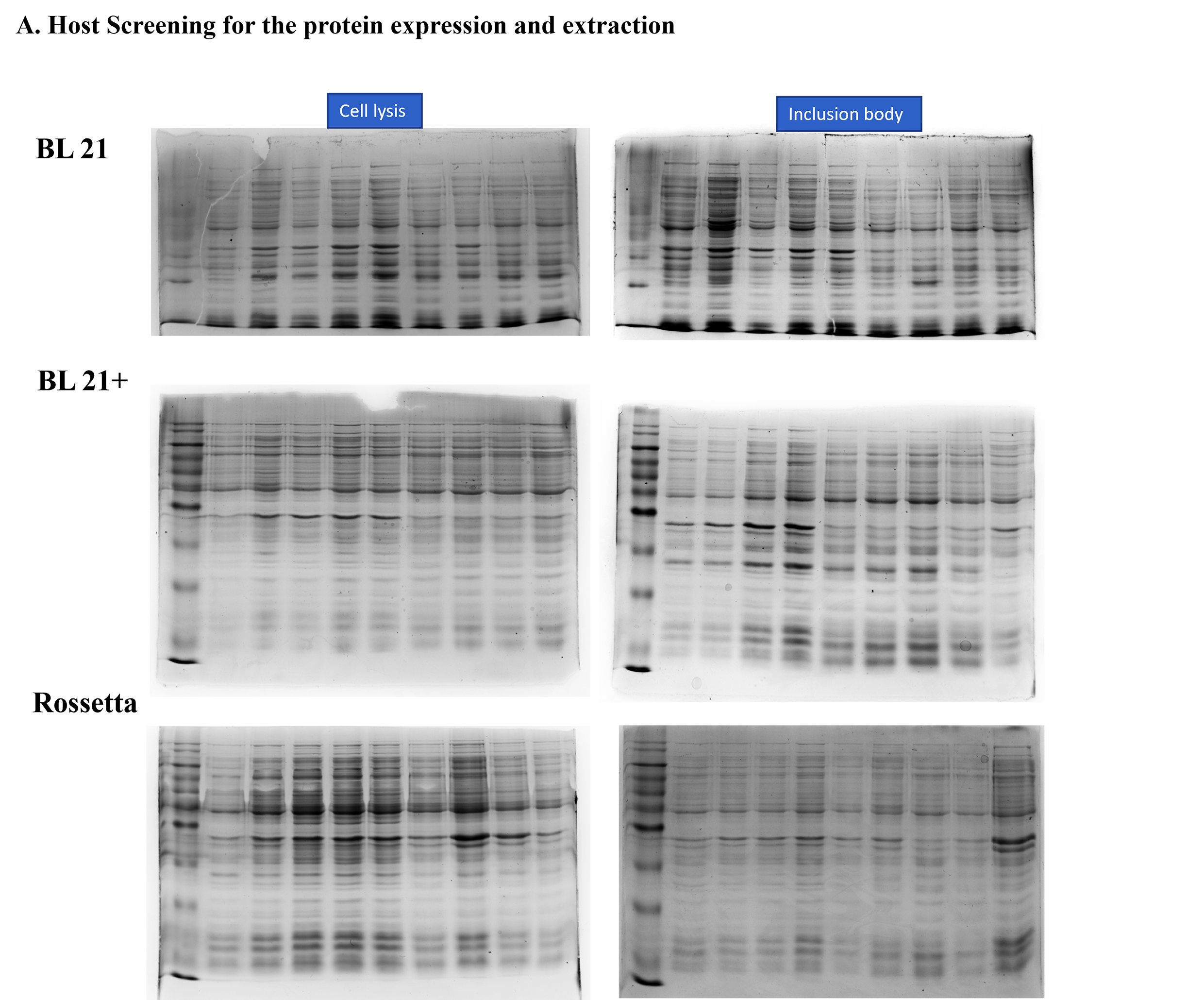

Supplement: Supplementary file 1 [file ijms-27-04304-s001.zip › Figure S6A.png]

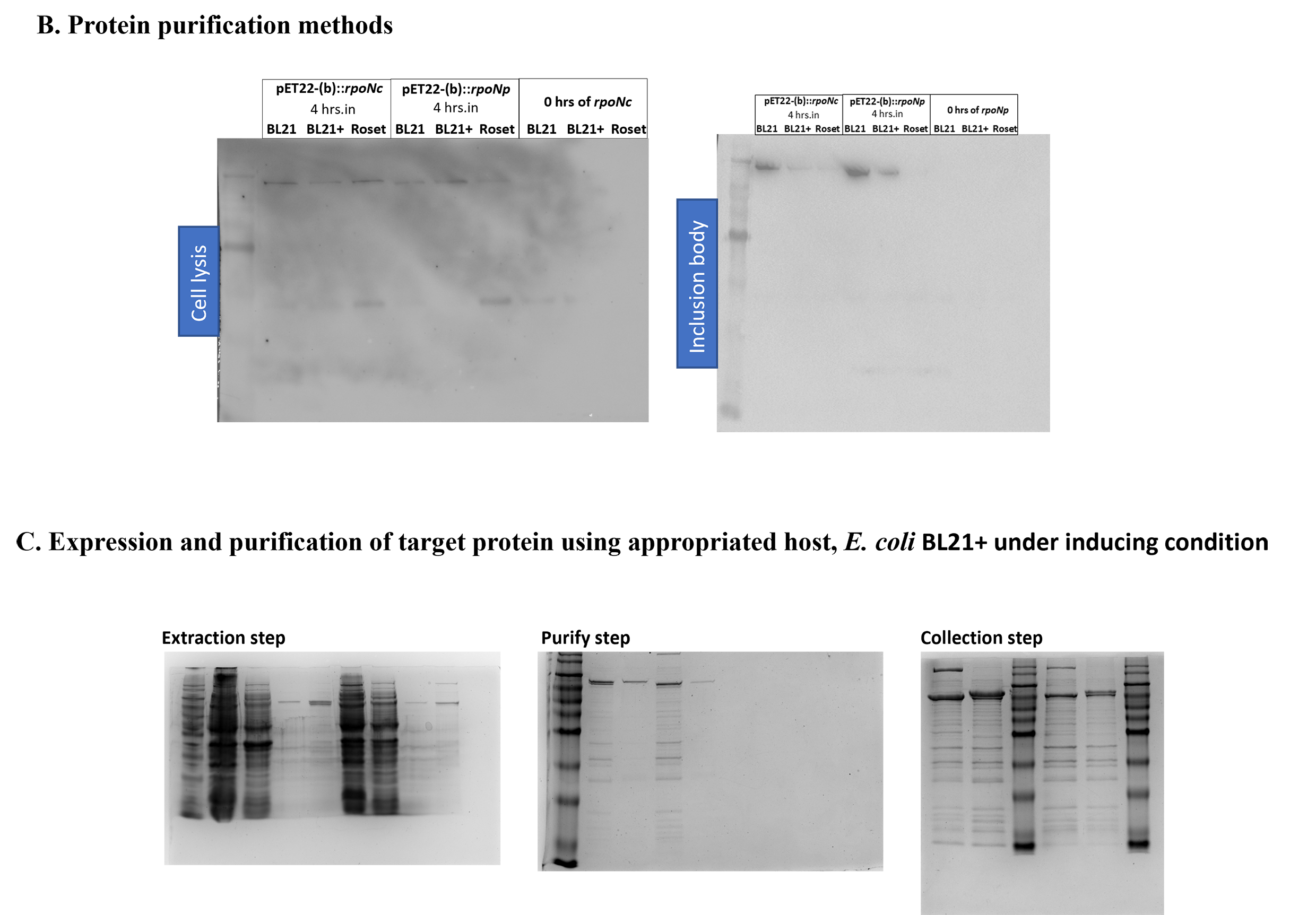

Supplement: Supplementary file 1 [file ijms-27-04304-s001.zip › Figure S6BC.png]

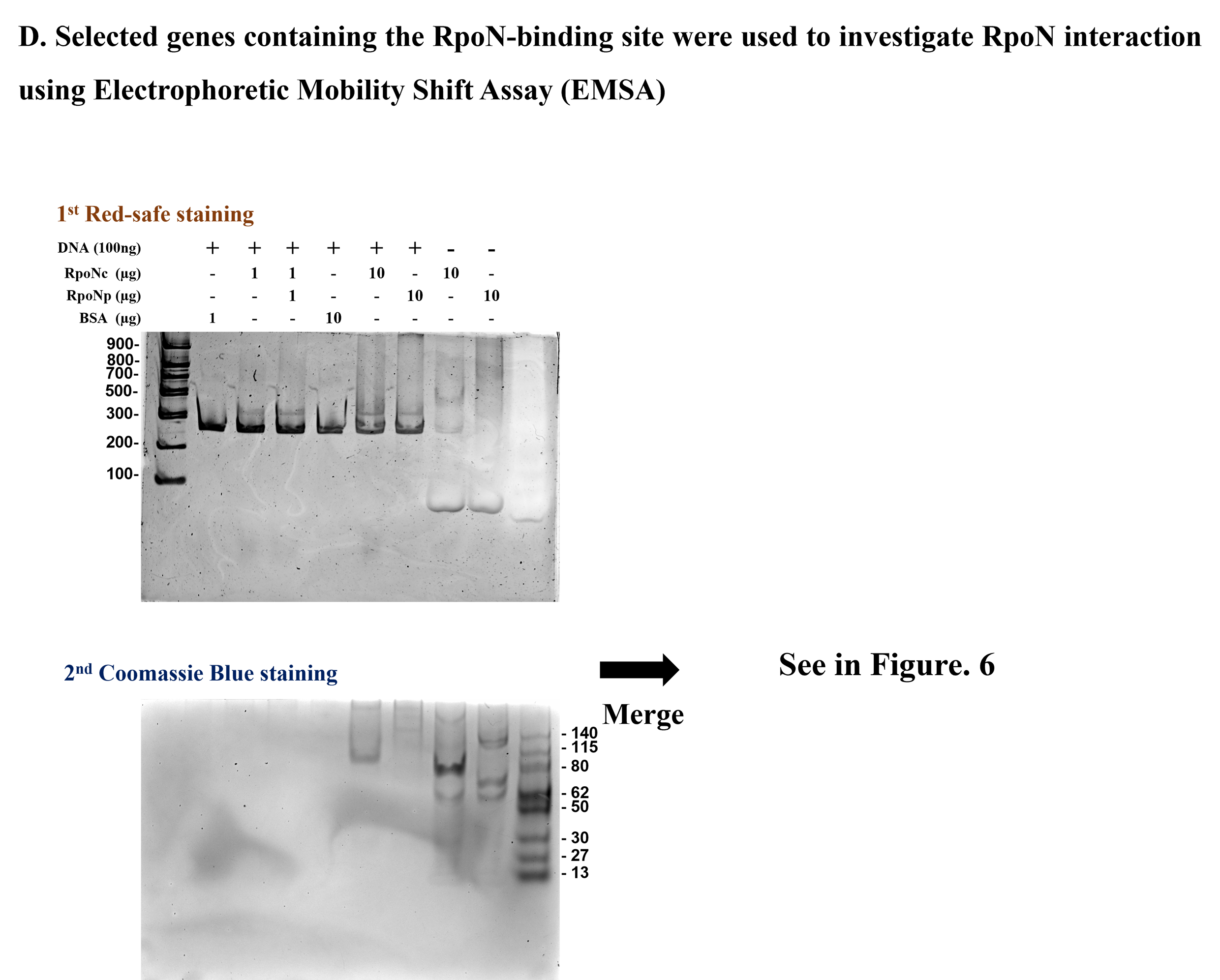

Supplement: Supplementary file 1 [file ijms-27-04304-s001.zip › Figure S6D.png]

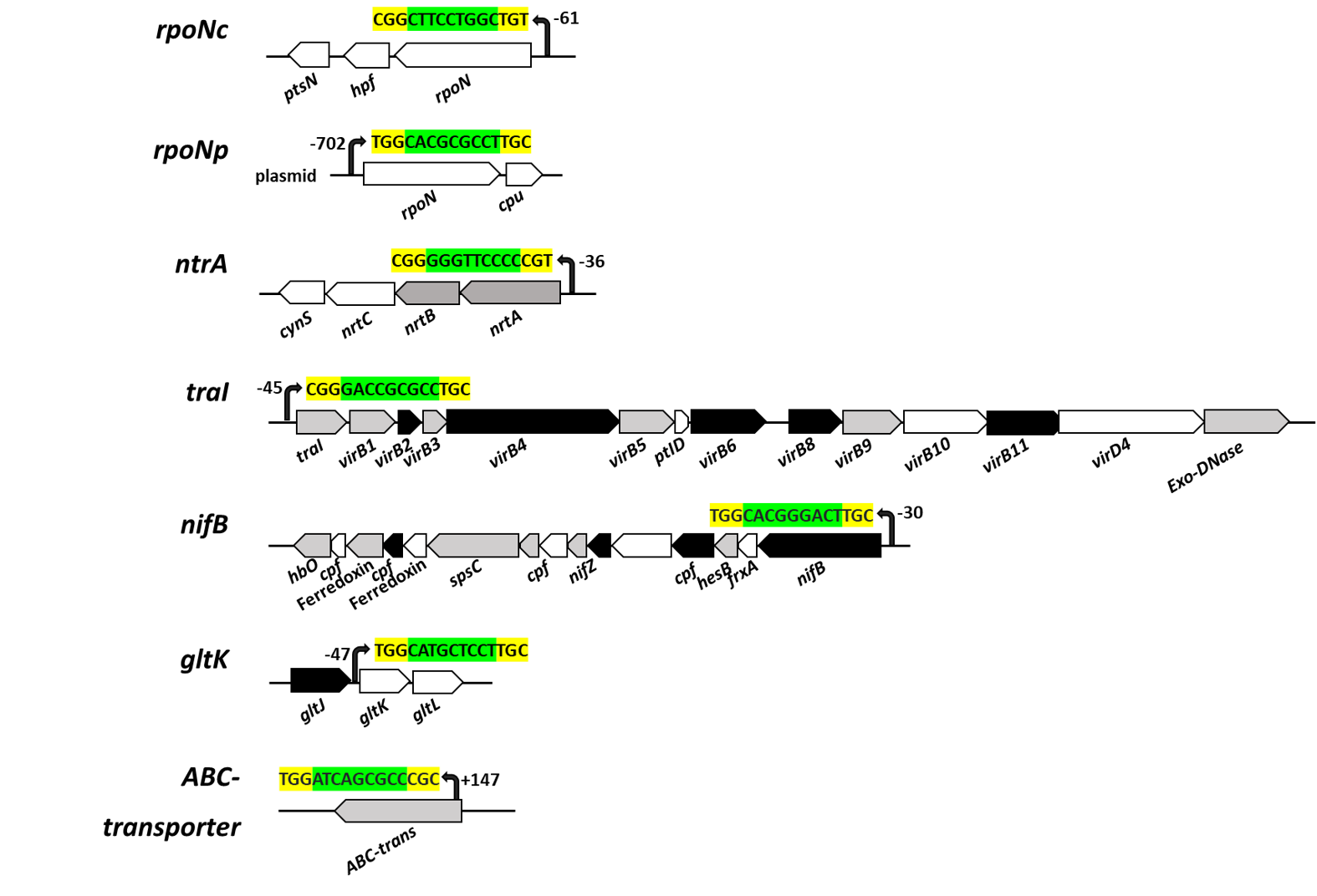

Supplement: Supplementary file 1 [file ijms-27-04304-s001.zip › Figure S7.png]
